# QTL Analysis of High Thermotolerance with Superior and Downgraded Parental Yeast Strains Reveals New Minor QTLs and Converges on Novel Causative Alleles Involved in RNA Processing

**DOI:** 10.1371/journal.pgen.1003693

**Published:** 2013-08-15

**Authors:** Yudi Yang, Maria R. Foulquié-Moreno, Lieven Clement, Éva Erdei, An Tanghe, Kristien Schaerlaekens, Françoise Dumortier, Johan M. Thevelein

**Affiliations:** 1 Laboratory of Molecular Cell Biology, Institute of Botany and Microbiology, KU Leuven, Flanders, Belgium; 2 Department of Molecular Microbiology, VIB, Leuven-Heverlee, Flanders, Belgium; 3 Department of Applied Mathematics, Computer Science and Statistics, Ghent University, Flanders, Belgium; EMBL, Germany

## Abstract

Revealing QTLs with a minor effect in complex traits remains difficult. Initial strategies had limited success because of interference by major QTLs and epistasis. New strategies focused on eliminating major QTLs in subsequent mapping experiments. Since genetic analysis of superior segregants from natural diploid strains usually also reveals QTLs linked to the inferior parent, we have extended this strategy for minor QTL identification by eliminating QTLs in both parent strains and repeating the QTL mapping with pooled-segregant whole-genome sequence analysis. We first mapped multiple QTLs responsible for high thermotolerance in a natural yeast strain, MUCL28177, compared to the laboratory strain, BY4742. Using single and bulk reciprocal hemizygosity analysis we identified *MKT1* and *PRP42* as causative genes in QTLs linked to the superior and inferior parent, respectively. We subsequently downgraded both parents by replacing their superior allele with the inferior allele of the other parent. QTL mapping using pooled-segregant whole-genome sequence analysis with the segregants from the cross of the downgraded parents, revealed several new QTLs. We validated the two most-strongly linked new QTLs by identifying *NCS2* and *SMD2* as causative genes linked to the superior downgraded parent and we found an allele-specific epistatic interaction between *PRP42* and *SMD2*. Interestingly, the related function of *PRP42* and *SMD2* suggests an important role for RNA processing in high thermotolerance and underscores the relevance of analyzing minor QTLs. Our results show that identification of minor QTLs involved in complex traits can be successfully accomplished by crossing parent strains that have both been downgraded for a single QTL. This novel approach has the advantage of maintaining all relevant genetic diversity as well as enough phenotypic difference between the parent strains for the trait-of-interest and thus maximizes the chances of successfully identifying additional minor QTLs that are relevant for the phenotypic difference between the original parents.

## Author Summary

Most traits of organisms are determined by an interplay of different genes interacting in a complex way. For instance, nearly all industrially-important traits of the yeast *Saccharomyces cerevisiae* are complex traits. We have analyzed high thermotolerance, which is important for industrial fermentations, reducing cooling costs and sustaining higher productivity. Whereas genetic analysis of complex traits has been cumbersome for many years, the development of pooled-segregant whole-genome sequence analysis now allows successful identification of underlying genetic loci with a major effect. On the other hand, identification of loci with a minor contribution remains a challenge. We now present a methodology for identifying minor loci, which is based on the finding that the inferior parent usually also harbours superior alleles. This allowed construction for the trait of high thermotolerance of two ‘downgraded parent strains’ by replacing in each parent a superior allele by the inferior allele from the other parent. Subsequent mapping with the downgraded parents revealed new minor loci, which we validated by identifying the causative genes. Hence, our results illustrate the power of this methodology for successfully identifying minor loci determining complex traits and with a high chance of being co-responsible for the phenotypic difference between the original parents.

## Introduction

Many genetic traits are quantitative and show complex inheritance. Because these traits are so prevalent in nature, understanding the underlying factors is important for various biological fields and for applications like industrial biotechnology and agricultural practice [1]. Recently, baker's yeast *Saccharomyces cerevisiae* has become an important subject for studies in quantitative genetics [2], [3]. In particular the availability of high-density genetic markers, the ease of performing experimental crosses and the powerful technologies for precise genetic modification [4], [5], do not only allow efficient QTL mapping but also rapid identification of causative genes and their experimental validation and interaction analysis. *S. cerevisiae* displays many quantitative traits that are also important in other cell types, including industrial microorganisms and cells of higher, multicellular organisms. Such properties include thermotolerance [6] and oxidative stress tolerance [7], the capacity to produce small molecules, such as acetic acid [8] and ethanol tolerance [9], [10]. Other quantitative traits that have been studied in yeast include transcriptional regulation [11], sporulation efficiency [12], telomere length [13], cell morphology traits [14], mitochondrial genome instability [15], global gene expression [16], evolution of biochemical pathways [17] and resistance to chemicals [18].

A major remaining challenge in quantitative trait studies is the efficient mapping of minor quantitative trait loci (QTLs) and identification of their causative genes. Minor QTLs have a subtle influence on the phenotype, which is easily masked by epistasis [19], gene-environment interactions [20], low association to the phenotype because of limited sample size and complex interactions with other QTLs. Minor QTLs are important because together they can produce in an additive or synergistic manner equally dramatic effects on the phenotype as major QTLs. Actually, the work of Bloom et al. [21], in which a large panel of individually genotyped and phenotyped yeast segregants was used, has shown that for 46 quantitative traits, the assembly of all detected loci could explain nearly the entire additive contribution to the heritable variation. The minor QTLs identified should be truly relevant for the trait of interest in the original parent strains and not generated in some unrelated way during the mapping procedure.

Several methods have been reported to identify minor QTLs. Sinha et al. [22] used a targeted backcross strategy to first eliminate a major QTL. Subsequent mapping revealed a novel allele that had an epistatic interaction with the first major QTL. A disadvantage of backcrossing is the reduction of genetic diversity, which likely leads to loss of minor QTLs. In a different approach, Lorenz and Cohen [23] fixed major QTLs either in the superior parent or in the inferior parent and successfully identified minor QTLs by linkage analysis by repeating the QTL mapping with the new parent strains. A potential problem caused by elimination of major QTLs in one parent is that the phenotypic difference between the two parent strains is reduced. This may make it more difficult to evaluate the phenotype of the extreme segregants in comparison with the superior parent. Parts et al. [24] used many millions of segregants and multiple inbreeding steps to facilitate the detection of statistically significant minor QTLs. The use of such a high number of segregants, however, is only feasible for selectable phenotypes. Swinnen et al. [10] made use of more stringent phenotyping, i.e. tolerance to higher ethanol levels, which revealed several additional minor QTLs. The disadvantage of this approach is that higher stringency of phenotyping requires higher numbers of segregants to be phenotyped to obtain enough segregants with the superior phenotype. In the study of Bloom et al. [21], aimed at identifying the source of missing heritability, linkage analysis was performed with a large panel of individually genotyped and phenotyped yeast segregants, which enabled detection of many QTLs with a small effect.

In this work we have extended previous approaches to identify minor QTLs to QTL mapping by pooled-segregant whole-genome sequence analysis and we eliminated the effect of major QTLs in both parents. Our method is based on the observation that superior haploid segregants of natural or industrial diploid strains usually contain mutations that to some extent compromise rather than promote the trait of interest. As a result genetic mapping with such segregants usually reveals QTLs, which are linked to the inferior parent rather than to the superior parent. This allows the construction of two new parent strains, which are both downgraded for the trait of interest by replacement of a superior allele with an inferior allele from the other parent. This maintains a large phenotypic difference between the new parent strains. They also retain all genetic diversity, in particular all remaining minor QTLs. We show the effectiveness of this approach by first mapping QTLs involved in high thermotolerance of a selected yeast strain compared to a control strain, identifying causative genes linked to the superior and inferior parent, constructing two downgraded parent strains and repeating the genetic mapping with the new parents. This revealed several new minor QTLs, which we validated by identifying the causative gene in two QTLs. Interestingly, the two novel causative genes identified in this study are both involved in pre-mRNA splicing, which suggests an important role for RNA processing in conferring high thermotolerance.

## Results

### Identification of QTLs determining high thermotolerance

**Figure 1:**
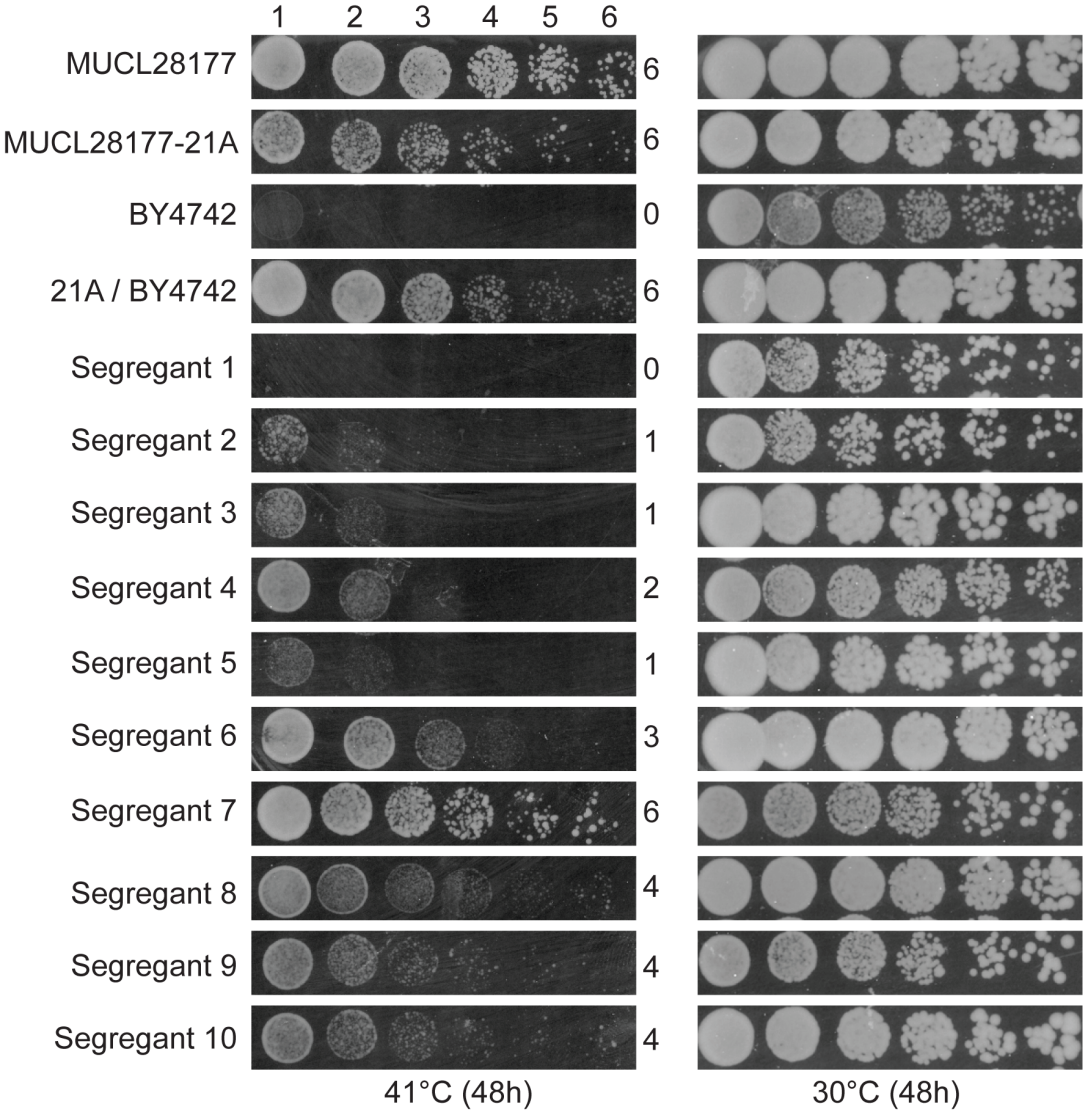
Thermotolerance of the parent strains and segregants. The diploid strain MUCL28177 was identified as a highly thermotolerant strain, showing strong growth at 41°C. One of its haploid segregants MUCL28177-21A (referred to as 21A) also showed high thermotolerance, whereas the control laboratory strain BY4742 did not grow at all at 41°C. The hybrid diploid strain 21A/BY4742 grew nearly as well at 41°C as its superior parent 21A, indicating that the major causative allele(s) in 21A is (are) dominant. The haploid segregants from 21A/BY4742 show varying growth ability (as indicated by a score from 0 to 6 for growth in the different dilutions) at 41°C, between that of the BY4742 inferior and 21A superior parents, indicating that thermotolerance is a quantitative trait.

We have screened a total of 305 natural and industrial isolates of *S. cerevisiae* for their ability to grow at high temperature, i.e. 40–41°C, on solid YPD plates. Not a single yeast strain was able to grow with a reasonable rate at 42°C. The strain MUCL28177 showed very good growth at 41°C and was chosen for further analysis. After sporulation, we selected a haploid segregant MUCL28177-21A, further referred to as 21A, which also showed excellent growth at 41°C compared to the control strain BY4742. Strain 21A was crossed with the laboratory strain BY4742, that is unable to grow at 41°C. The hybrid 21A/BY4742 diploid strain grew at least as well as the 21A strain at 41°C, indicating that the high thermotolerance of 21A is a dominant characteristic. Phenotyping of 950 segregants of the 21A/BY4742 diploid strain revealed a range of thermotolerance. It resulted in 58 segregants with similar growth at high temperature as 21A. The growth of the original strain MUCL28177, the parent strains 21A and BY4742, the hybrid diploid strain 21A/BY4742 and ten representative segregants with varying thermotolerance, is shown in Figure 1.

**Figure 2:**
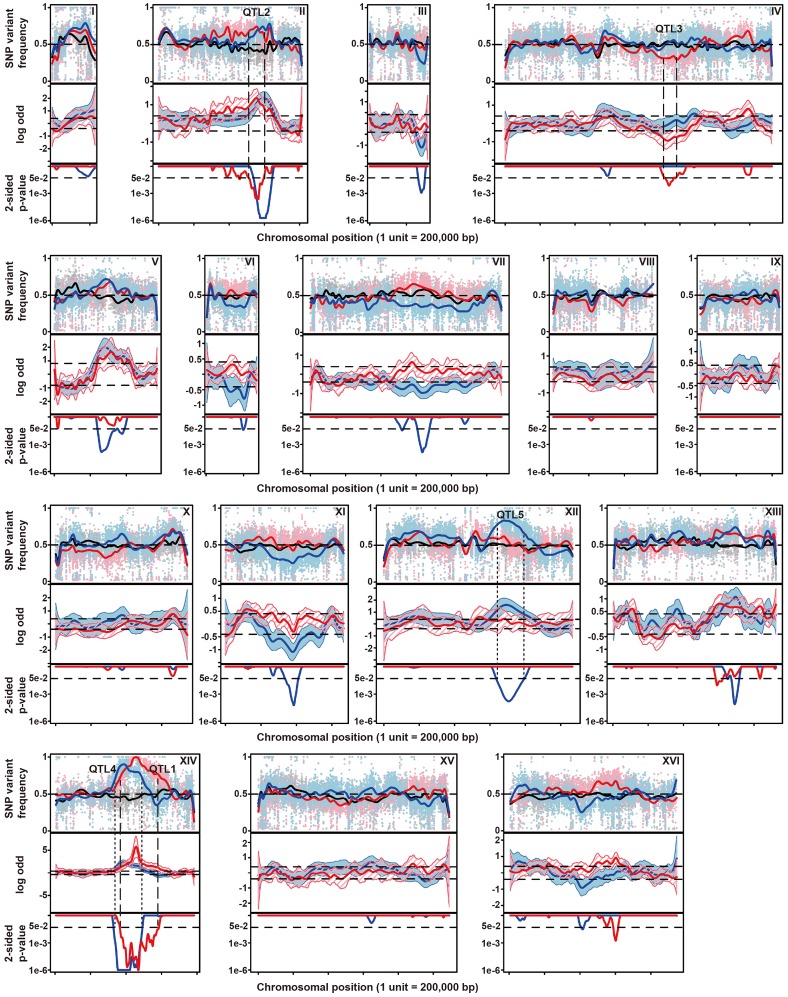
Genetic mapping of QTLs involved in thermotolerance by pooled-segregant whole-genome sequence analysis. Genomic DNA samples were extracted from an unselected pool (pool 0) and two pools of thermotolerant segregants able to grow at 41°C (Pool 1) and at 40.7°C (Pool 2), respectively. The DNA for each pool originates from 58 segregants. Pool 1 consists of segregants from the cross between parents 21A and BY4742 and Pool 0 and Pool 2 from the cross between the downgraded parents 21A^DG^ and BY4742^DG^. The top-panel represents the SNP variant frequency of pool 0 (small gray circles), pool 1 (small pink triangles) and pool 2 (small blue diamonds) along with the smoothed SNP frequency profile (black line: pool 0, red line: pool 1 and blue line: pool 2) using a generalized additive model. In the middle panel the log odds ratio (contrast) between the SNP variant frequency of a selected pool and pool 0 is plotted along with simultaneous 95% confidence bands (red region: pool 1 and blue region: pool 2). Horizontal dash lines indicate the threshold (δ = 0.4088). The bottom panel shows 2-sided p-values along the chromosome that are corrected for multiple testing, with horizontal dash lines indicating a cut-off of 0.05. Confirmed QTLs are indicated at corresponding positions, with broken lines indicating QTLs from the original parents, and stippled lines QTLs from the downgraded parents.

The 58 thermotolerant segregants were pooled based on dry weight and genomic DNA isolated from the pool. Genomic DNA samples from the pooled segregants and from parent strain 21A were sequenced. The sequence reads obtained were aligned with the sequence of the reference S288c genome, which is essentially the same as that of the inferior parent strain BY4742. A set of quality-filtered SNPs to be used as genetic markers, was acquired essentially as described before [10]. For each chromosome, the SNP variant frequency was modeled using an additive logistic regression model [10], [25]. The results are shown in Figure 2. In the top panel, the raw SNP frequencies are plotted against the chromosomal position along with the modeled frequency (smoothed lines). The middle panel shows contrasts between selected pools and an unselected pool along with 95% simultaneous confidence bands. Upward and downward deviations from 0 indicate putative QTLs containing causative alleles from the superior and inferior parent, respectively. Normally, only linkage with the superior parent strain is expected. However, since the original MUCL28177 diploid strain is a natural isolate, it is likely heterozygous. Hence, the 21A segregant may contain recessive mutations that compromise to some extent thermotolerance in spite of the fact that its overall thermotolerance was only slightly lower than that of the MUCL28177 parent strain.

We calculated 2-sided p-value profiles along the chromosome that were adjusted for multiple testing (Text S1 online: Supplementary Methods) and five regions show significant p-values (0.05 significance level, Figure 2). We chose four regions with the smallest p-values for further analysis (Table S1 online). For these loci, selected SNPs were scored in individual thermotolerant segregants (up to 62 after additional segregant isolation and phenotyping) and a binomial exact test with FDR adjusted p-values was used for assessing statistical significance [10], [26]. Three QTLs (QTL1, QTL2 and QTL3) were confirmed to exhibit statistically significant linkage to the high thermotolerance phenotype (0.05 FDR level, Table 1). QTL1 and QTL2 showed linkage with the genome of the superior 21A parent strain, while QTL3 showed linkage with the genome of the inferior BY4742 parent strain. We concentrated our work first on QTL1 and QTL3, because they showed the strongest linkage to the superior and inferior parent, respectively. The subtelomeric regions often show deviations from the 50% value of the SNP variant frequency, but this is also observed in the unselected pool. It may be caused by complications with the mapping of repetitive sequences, which are known to be commonly present in subtelomeric regions. We have analysed for instance the right subtelomeric region of chromosome X, in the mapping with the original parents, using SNP detection in the individual segregants and found a p-value that failed to indicate significant linkage (results not shown).

**Table 1:** List of QTLs identified in the mapping with the original parents.

QTL	Location	Total number of thermotolerant segregants used	Association to superior parent strain 21A	FDR p-value
QTL1	435069–475213 on chromosome XIV	46	100%	5.16e-13
QTL2	540838–560167 on chromosome II	62	74.20%	1.60e-3
QTL3	949927–999889 on chromosome IV	62	29.03%	1.18e-2

### Identification of the causative gene in QTL1

**Figure 3:**
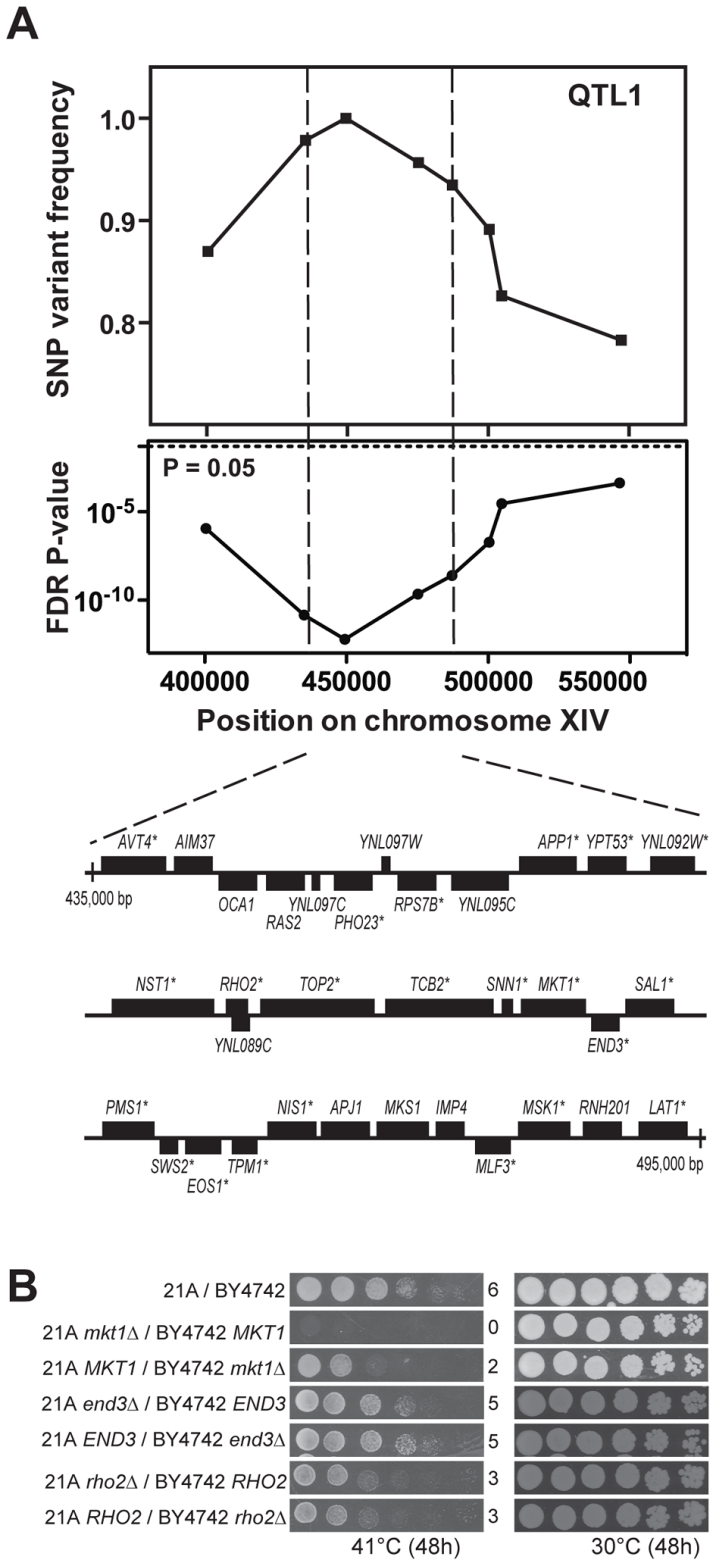
Dissection of QTL1 to identify the causative gene. (A) Fine-mapping of QTL1 by scoring selected SNPs in the individual thermotolerant segregants. Eight SNPs spanning between 400,000 bp and 550,000 bp on chromosome XIV were scored by PCR in 46 thermotolerant segregants and both SNP variant frequency and FDR p-value were calculated. A 60,000 bp region between SNP2 and 5 showed the strongest linkage. It contained 33 genes and putative ORFs as indicated using the annotations in SGD. The genes containing at least one non-synonymous mutation within the ORF are indicated with an asterisk. (B) Identification of the causative gene *MKT1* in QTL1. RHA results for *MKT1*, *RHO2* and *END3* in the central region of QTL1 are shown. The strain pairs for the same genes were always spotted on the same plate. The results for the original hybrid diploid 21A/BY4742 and the *MKT1* reciprocal deletion strains were also from the same plate.

We first fine-mapped QTL1 by scoring eight selected SNPs in individual thermotolerant segregants, which reduced the size of the locus to about 60,000 bp (Figure 3A). Detailed analysis of the 21A sequence of this region showed that 22 out of the 33 genes and putative ORFs present contained at least one non-synonymous mutation in the ORF compared to the BY4742 sequence (Figure 3A). Next we applied reciprocal hemizygosity analysis (RHA) [6] to identify causative gene(s) in QTL1. RHA is used to test for a possible contribution to the phenotype of each allele of the candidate gene in a hybrid genetic background. For each of the 22 genes with non-synonymous mutations, we constructed two 21A/BY4742 hybrid strains in which either the 21A or the BY4742 allele was deleted, so that each strain only contained one specific allele of the candidate gene. Comparison of the growth at high temperature (41°C) of the two hybrid strains did not show any difference for the 22 candidate genes, except for *MKT1* (Figure 3B, Figure S1 online and data not shown). The hybrid strain with the *MKT1^21A^* allele showed better growth than the strain with the *MKT1^BY4742^* allele. We further confirmed the relevance of *MKT1* by demonstrating that *MKT1* deletion reduced thermotolerance in the 21A strain background (Figure S2 online). Since 21A with either *mkt1Δ* or *MKT1^BY4742^* showed the same growth at 40.7°C and since BY4742 showed the same growth at 40.7°C as BY4742 *mkt1Δ*, the *MKT1^BY4742^* allele behaves as a loss of function allele for thermotolerance when assayed under our conditions and in our haploid strain backgrounds (Figure S2 online).

In a previous QTL mapping study of thermotolerance with a clinical isolate of *S. cerevisiae* and the lab strain S288c, the *MKT1* allele of the clinical isolate was also identified as a causative gene [6] and in a follow-up study, out of two polymorphisms in Mkt1, D30G and the conservative substitution K453R, the D30G mutation was identified as the causative mutation [27]. Sanger sequencing of *MKT1^21A^* confirmed that Mkt1-21A has the same mutations. *END3* and *RHO2*, which are located close to *MKT1* in the same QTL, were also reported to have an allele-specific contribution to thermotolerance [6]. However, in the current experimental setup, the *RHO2* alleles from our two genetic backgrounds did not produce a difference in thermotolerance, while for *END3* there may be a slight difference (Figure 3B). Sequence alignment using the Illumina sequencing data shows that *END3^21A^* lacks the causative SNP (C773T) found in *END3^YJM145^* [27]. In the case of *RHO2* it is known that SNPs in the 3′UTR of *RHO2^YJM145^* are responsible for the phenotypic effect on thermotolerance. *RHO2^21A^* contains the same SNPs in its 3′UTR except for insertion of an A six base pairs downstream of the ORF. Hence, this insertion in *RHO2^YJM145^* may cause the growth advantage at high temperature.

### Identification of the causative gene in QTL3

**Figure 4:**
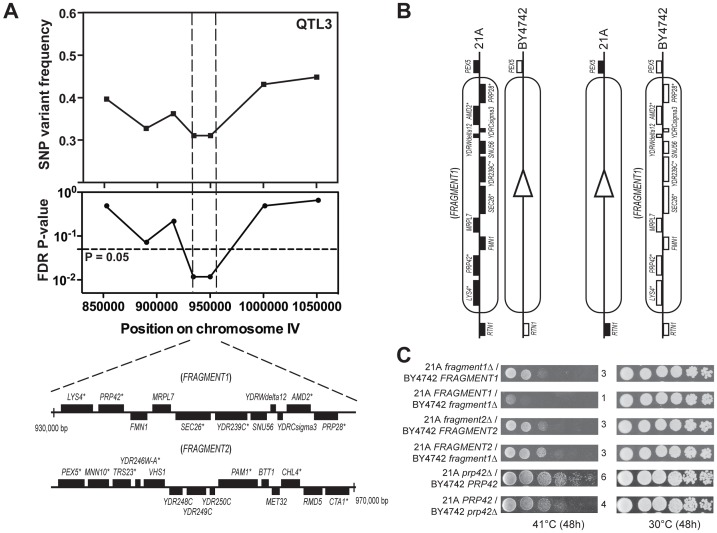
Dissection of QTL3 to identify the causative gene. (A) Fine-mapping of QTL3 by scoring seven selected SNPs in 62 individual thermotolerant segregants confirms significant linkage with the genome of the inferior parent strain BY4742 of the region between 930,000 and 970,000 bp on chromosome IV. The genes containing at least one non-synonymous mutation within the ORF are indicated with an asterisk. This region has been divided into two fragments for bulk RHA, as indicated. (B) Example of bulk RHA for the block of genes on *FRAGMENT1*. A pair of reciprocal deletion strains for either *FRAGMENT1* or *FRAGMENT2* was constructed as shown and tested for growth at high temperature. (C) Identification of the causative gene in QTL3. Bulk RHA shows that *FRAGMENT1*, derived from BY4742, confers higher thermotolerance compared to *FRAGMENT1*, derived from 21A, whereas for *FRAGMENT2* there was no difference. RHA for the individual genes within *FRAGMENT1* identified *PRP42* as the causative gene.

QTL3 is linked to the genome of the inferior parent strain, indicating that BY4742 contains a superior genetic element for thermotolerance in this region. We fine-mapped QTL3 by scoring seven selected SNPs in 62 thermotolerant segregants individually. This reduced the locus to 40,000 bp (Figure 4A). Detailed analysis of the 21A sequence in this region revealed 13 genes and putative ORFs with at least one non-synonymous mutation (Figure 4A).

To accelerate identification of the causative genes in this region, we first performed ‘bulk RHA’. Instead of comparing alleles for each single gene, we first made a reciprocal deletion in the hybrid strain of a fragment with multiple genes. We divided the 40,000 bp region of QTL3 into two fragments, with the first containing 11 genes and the second 14 genes (Figure 4A, 4B). For each fragment, we constructed two hybrid strains with one strain containing only the fragment from the 21A background and the other only the fragment from the BY4742 background (Figure 4B). Comparison of growth at high temperature (41°C) showed that *FRAGMENT1^BY4742^* conferred better growth at high temperature than *FRAGMENT1^21A^*, while there was a much smaller difference between the strains with *FRAGMENT2^BY4742^* or *FRAGMENT2^21A^* (Figure 4C).

We then applied RHA for the six individual genes of *FRAGMENT1* that had at least one non-synonymous mutation (Figure 4C). This identified *PRP42^BY4742^* as a superior allele for thermotolerance compared to *PRP42^21A^*, whereas for the other genes there was no allele-specific difference in thermotolerance (Figure S3 online). We also tested growth at high temperature of strains containing a heterozygous deletion of either the complete *FRAGMENT1* or only the *PRP42* gene together on the same plate. We found the growth at 41°C to be similar whether the complete *FRAGMENT1* or only the *PRP42* gene from either BY4742 or from 21A was deleted (Figure S4 online). This suggests that *PRP42* was likely the only causative gene in *FRAGMENT1* and thus also seems to exclude the other genes without non-synonymous mutation in their ORF as possible causative gene. As an additional control, we also performed RHA with the seven genes of Fragment 2 with a non-synonymous mutation in their ORF and we did not find any difference between the alleles from the two parent strains in conferring thermotolerance (data not shown).

*PRP42^21A^* has eleven mutations compared to *PRP42^BY4742^*, with three of them being non-synonymous and the other eight synonymous (Table S2 online). The three polymorphisms in Prp42, H296Y, F467S, and E526Q, are non-conservative substitutions, but it is difficult to predict a possible effect on the function or structure of the protein. They are located in domains without strong conservation (data not shown). Since no mutation was present in the promoter and terminator region, the difference in thermotolerance conferred by the two *PRP42* alleles is likely due to the change in protein sequence and thus in functionality. We have investigated the presence of these mutations in 22 other yeast strains, isolated from various sources, and of which the whole genome has been sequenced (Table S2 online), and found that among the three non-synonymous mutations, C886T is unique to 21A, whereas the other two mutations (C1400T and C1576G) are present in all other strains except in the lab strains S288c, CEN.PK113-7D and W303. If we assume that the inferior *PRP42* allele is rare (like the inferior *MKT1* allele in S288c), then C886T is the best candidate for the causative mutation. On the other hand, we cannot exclude that C886T is only one of the causative mutations, that it requires interaction with one or more of the other mutations or that a combination of the other SNPs is causative for the phenotype.

### Construction and phenotyping of the downgraded parent strains

**Figure 5:**
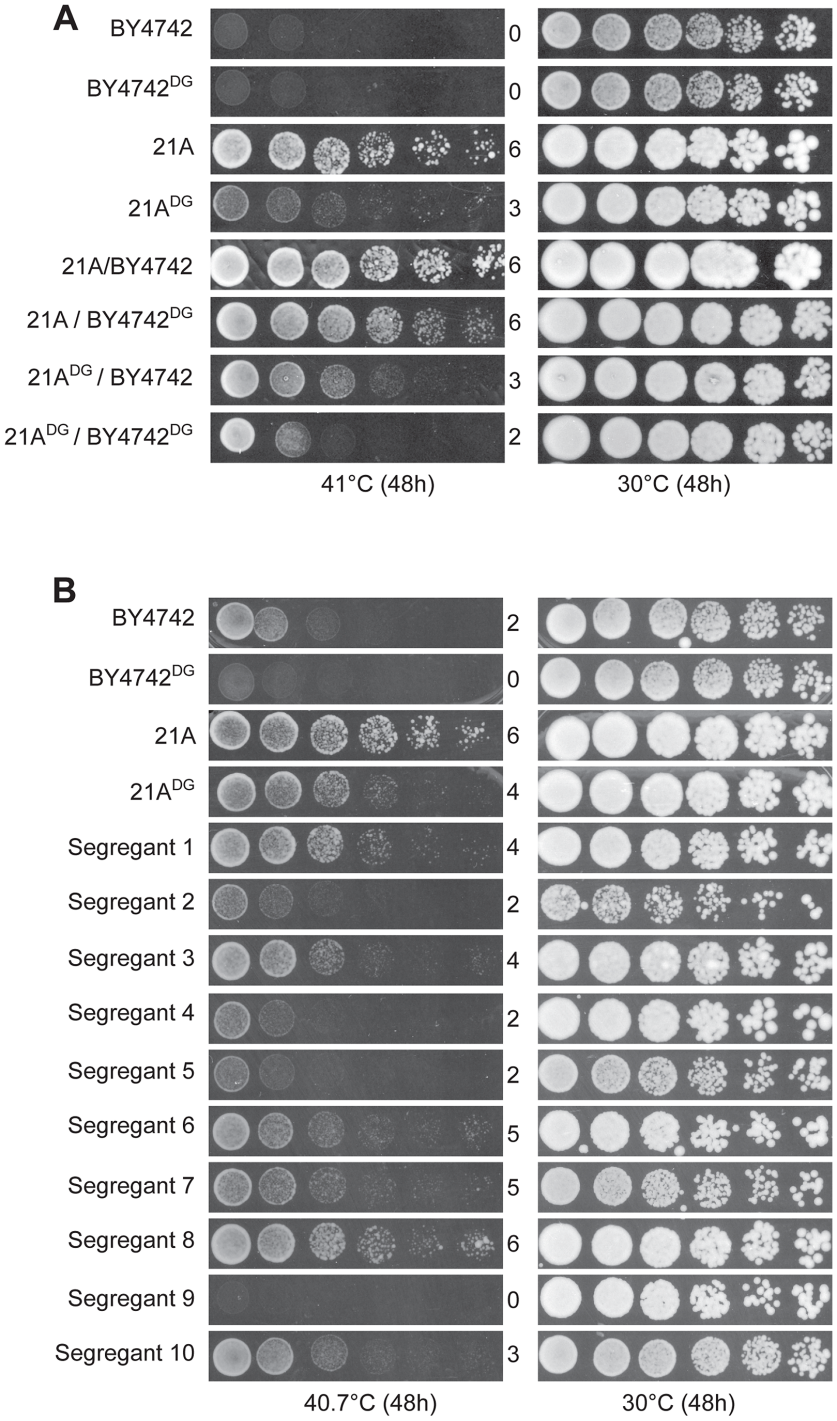
Thermotolerance of the downgraded parent strains and their segregants. (A) Growth at 41°C of the original parent strains, 21A and BY4742, the downgraded parent strains, 21A^DG^ and BY4742^DG^, and hybrid diploids in the four combinations. All strains were spotted on the same plate. (B) Growth at 40.7°C of the original parent strains, 21A and BY4742, the downgraded parent strains, 21A^DG^ and BY4742^DG^, and ten segregants from the hybrid 21A^DG^/BY4742^DG^. The strain pairs for each gene were always spotted on the same plate.

We next constructed two downgraded parent strains each with their own superior allele replaced by the inferior allele of the other parent: 21A^DG^: 21A *mkt1Δ:: MKT1^BY4742^* and BY4742^DG^: BY4742 *prp42Δ::PRP42^21A^*. Growth at 41°C of 21A^DG^ was reduced compared to 21A, confirming the importance of *MKT1^21A^* for high thermotolerance in 21A (Figure 5A). At 41°C, BY4742 and also BY4742^DG^ are not able to grow (Figure 5A). Hence, we reduced the temperature to 40.7°C, which allowed to demonstrate reduced growth of BY4742^DG^ compared to BY4742 (Figure 5B). Also at 41°C, we could demonstrate the beneficial effect of *PRP42^BY4742^* compared to *PRP42^21A^* by comparing growth of the 21A^DG^/BY4742 and 21A^DG^/BY4742^DG^ hybrid strains (Figure 5A). The availability of the four hybrid diploid strains also allowed us to demonstrate that in this background the effect of the *MKT1* and *PRP42* genes on thermotolerance is independent. The hybrid diploids, 21A^DG^/BY4742 and 21A/BY4742^DG^, each with replacement of one superior allele, both showed reduced growth at 41°C compared to the original hybrid of the parent strains, 21A/BY4742, while the hybrid of the two downgraded parent strains, 21A^DG^/BY4742^DG^, in which both superior alleles are replaced, showed further reduced growth (Figure 5A). (In this figure all strain pairs were put on the same plate.)

### Isolation and phenotyping of segregants from the downgraded parent strains

Figure 5 shows that both at 41°C and 40.7°C, the two downgraded parent strains, 21A^DG^ and BY4742^DG^, still show a strong difference in thermotolerance. We sporulated the 21A^DG^/BY4742^DG^ diploid strain and phenotyped 2464 segregants for thermotolerance. Examples are shown in Figure 5B. The segregants showed a range of thermotolerance and also transgressive segregation [28], since some of the segregants showed poorer thermotolerance than the inferior BY4742^DG^ parent (e.g. segregant 9 in Figure 5B) while others showed better thermotolerance than the superior 21A^DG^ parent (e.g. segregant 8 in Figure 5B). This suggests the presence of additional QTLs and causative genes influencing thermotolerance.

### Identification of new QTLs with segregants from the downgraded parents

From the 2464 segregants derived from the diploid 21A^DG^/BY4742^DG^ we selected 58 thermotolerant segregants that grew at 40.7°C at least as well as the 21A^DG^ superior parent strain, and repeated the pooled-segregant whole-genome analysis. We have used the same set of SNPs as generated in the previous sequencing of the 21A parent strain compared to S288c, for the mapping of QTLs linked to thermotolerance. A total of ten regions have a 2-sided p-value low enough for significance (Figure 2). Interestingly, two regions can be discerned with a clear difference between the original and downgraded pool (Figure 2, Table S1 online). The previous peak indicating linkage of one or more causative elements in the region between about 400,000 bp and 600,000 bp on chromosome XIV with the superior parent 21A (QTL1) has shifted to a more upstream position in the mapping with the 21A^DG^ downgraded superior parent (QTL4). In the region between 600,000 bp and 800,000 bp on chromosome XII, there is a new conspicuous peak, indicating linkage with the 21A^DG^ superior parent (QTL5). We confirmed the statistical significance of these two new QTLs by scoring selected SNPs in the individual thermotolerant segregants and performing a binomial exact test (Table 2). For the remaining seven regions, the SNPs showed about 50% variant frequency in the unselected pool (Figure S5 online). This suggests that the putative weak linkage from these regions is not caused by allelic incompatibilities. In addition, the significant association of the causative element(s) in QTL3 with the inferior parent (71% of the segregants had the genotype of the inferior parent, as determined by individual segregant genotyping) observed in the first mapping was completely abolished in the second mapping (52% of the segregants had the genotype of the inferior parent), which reaffirms that *PRP42* is the only causative gene in this locus.

**Table 2:** List of QTLs identified in the mapping with the downgraded parents.

QTL	Location	Total number of thermotolerant segregants used	Association to downgraded superior parent strain 21A^DG^	FDR p-value
QTL4	361531–392591 on chromosome XIV	58	91.38%	1.92e-10
QTL5	619142–791000 on chromosome XII	58	75.86%	4.92e-4

### Identification of causative genes in the new QTL4 and QTL5

In a previous QTL mapping study of thermotolerance [22], the authors identified the *NCS2* allele of a clinical isolate as a superior allele compared to the inferior allele from the S288c control strain. Since *NCS2* is located in the central region of QTL4 and since the *NCS2^21A^* allele contains the same mutation (A212T) as identified in the previous study, we have tested whether *NCS2^21A^* is also a causative allele in our genetic background. For that purpose, we performed RHA for *NCS2* using a hybrid diploid strain constructed from the two downgraded parent strains. We found that the *NCS2^21A^* allele supported higher thermotolerance compared to the *NCS2^BY4742^* allele, indicating that also in our genetic background the *NCS2* allele from the superior strain acted as a causative gene (which does not preclude the presence of other causative genes). Deletion of the inferior *NCS2^BY4742^* allele in the hybrid diploid strain also caused a conspicuous drop in thermotolerance (Figure S6 online).

**Figure 6:**
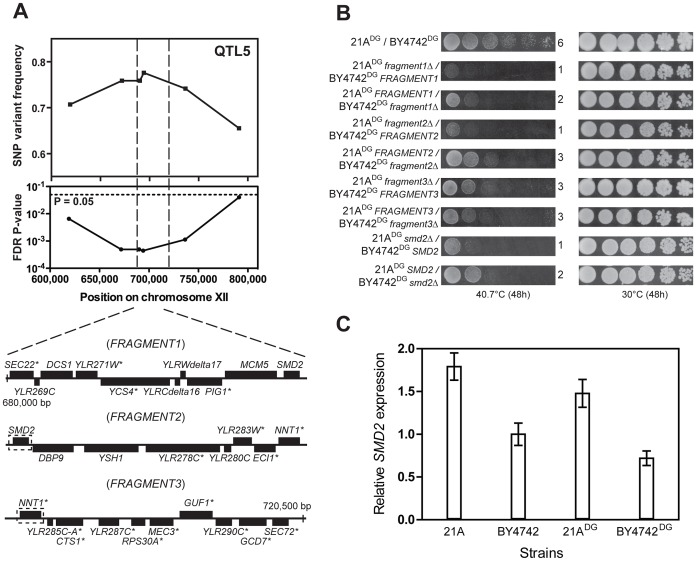
Dissection of QTL5 to identify the causative gene. (A) Fine-mapping of QTL5 by scoring six selected SNPs in 58 individual thermotolerant segregants confirms significant linkage of the region between 625,000 and 780,000 bp on chromosome XII to the genome of the superior 21A^DG^ parent strain. The region between 680,000 and 720,500 bp, showing the strongest linkage, was analysed for causative gene(s) as annotated in SGD. The genes containing at least one non-synonymous mutation within the ORF are indicated with an asterisk. This region was divided into three fragments for bulk RHA, as indicated. Overlapping genes in successive fragments (*SMD2* and *NNT1*) are indicated with a stippled box. (B) Bulk RHA with the three fragments. *FRAGMENT1* and *FRAGMENT2* from the 21A^DG^ parent strain confer higher thermotolerance at 40.7°C than the corresponding fragment from BY4742^DG^, while for *FRAGMENT3* there was no difference. RHA confirmed that *SMD2^21A^* conferred higher thermotolerance compared to the *SMD2^BY4742^*. (C) *SMD2* expression level as measured by QPCR in strains 21A, BY4742, 21A^DG^ and BY4742^DG^. The expression level in BY4742 was set equal to 1. Samples were taken at mid-exponential phase at 30°C.

Fine-mapping of QTL5 by scoring six selected SNPs individually in all 58 thermotolerant segregants enabled us to reduce the size of the QTL from 150,000 bp to 40,000 bp (Figure 6A). We then divided this region into three fragments and performed bulk RHA with each fragment in the 21A^DG^/BY4742^DG^ diploid strain (Figure 6A). (The fragments had an overlap of one gene.) Evaluation of thermotolerance with the pairs of reciprocally deleted hemizygous strains revealed that *FRAGMENT1^21A^* and *FRAGMENT2^21A^* conferred higher thermotolerance than the corresponding fragments from the inferior BY4742^DG^ parent. For *FRAGMENT3* there was no difference (Figure 6B). We then performed RHA with all individual genes of Fragments 1 and 2 containing non-synonymous mutations in their ORF (as indicated in Figure 6A). However, for none of the genes tested there was a different effect on thermotolerance of the two alleles (data not shown). We then applied RHA to the remaining genes in *FRAGMENT2* and found that the *SMD2^21A^* allele conferred higher thermotolerance compared to the *SMD2^BY4742^* allele (Figure 6B). Hence, it apparently acted as a causative allele in both *FRAGMENT1* and *FRAGMENT2*, since it was the only gene present in the overlap between the two fragments. The observation that replacement of *FRAGMENT1^21A^* with *FRAGMENT1^BY4742^* caused a similar reduction in thermotolerance compared to the replacement of *FRAGMENT2^21A^* with *FRAGMENT2^BY4742^* is consistent with *SMD2* being the only causative gene in QTL5.

We confirmed by Sanger sequencing that *SMD2^21A^* only displayed SNPs in the promoter and terminator region as compared to *SMD2^BY4742^* (data not shown). Hence, a difference in expression level may be responsible for the difference in thermotolerance. We have compared *SMD2* transcription levels in different strains and with incubation at different temperatures. We found a higher level of *SMD2* expression for 21A compared to BY4742 in cells growing exponentially in liquid cultures (YPD at 30°C) and also 21A^DG^ showed a higher level of *SMD2* expression under these conditions than BY4742^DG^ (Figure 6C). The difference in *SMD2* expression level is also clear for the 21A/BY4742 RHA pairs, but there is no significant difference for the 21A^DG^/BY4742^DG^ RHA pairs (Table S3 online). This indicates that the mechanism of *SMD2* in influencing thermotolerance cannot be solely due to differences in its transcript level, and other mechanisms such as post-transcriptional regulation may play a role.

### Detection of an allele-specific epistatic interaction between *PRP42* and *SMD2*

**Figure 7:**
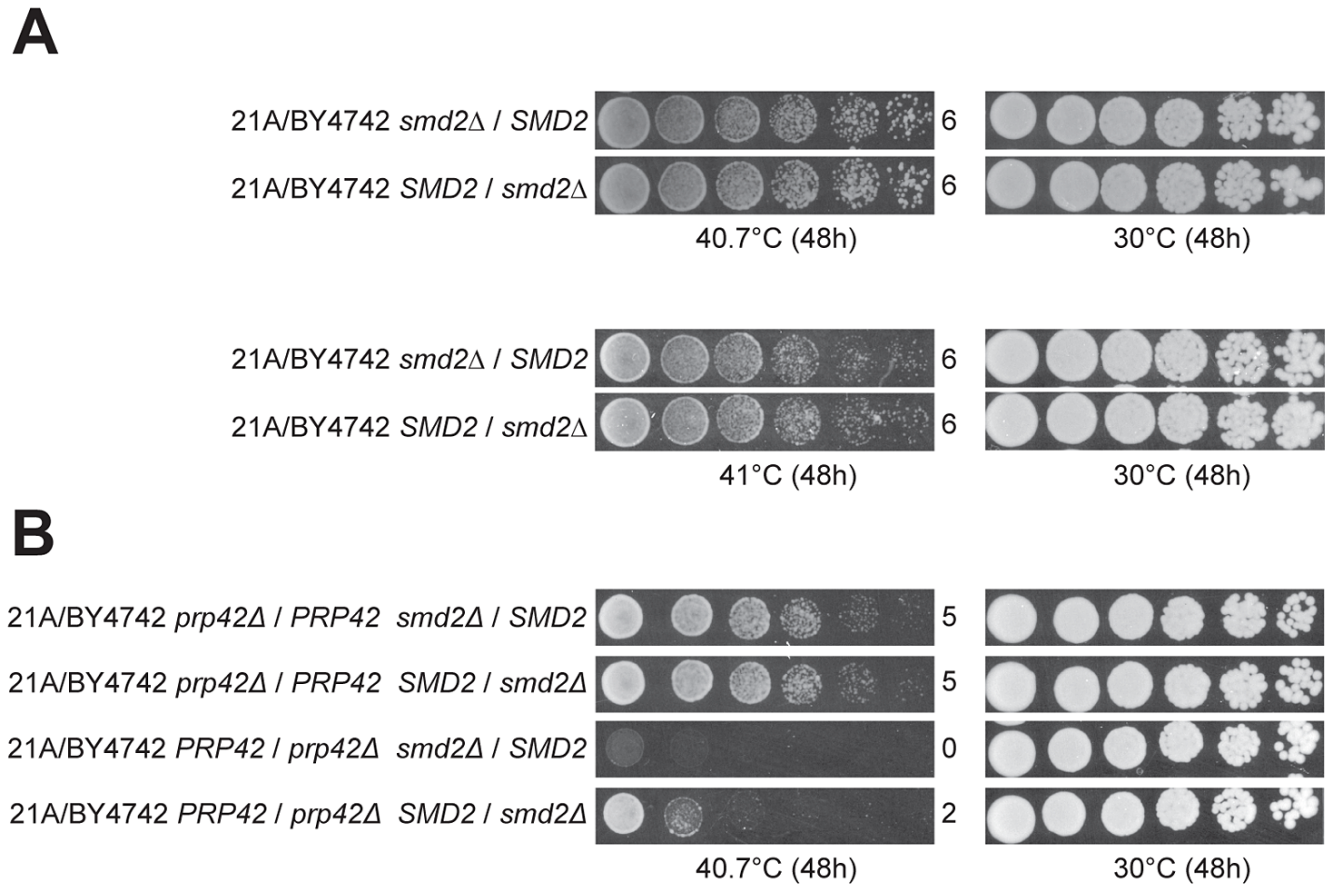
Detection of an allele-specific interaction between *PRP42* and *SMD2*. (A) RHA for *SMD2* in the hybrid strain made with the original parents, 21A/BY4742, failed to reveal any difference in thermotolerance either at 40.7 or 41°C conferred by the two *SMD2* alleles. (B) RHA for double heterozygous deletion of *PRP42* and *SMD2* shows that the effect of *SMD2^21A^* is dependent on the presence/absence of *PRP42^BY4742^*.

In the cross with the original parents, the QTL5 region did not show any indication of linkage to the genome of the superior parent strain 21A, with 37 out of 58 thermotolerant segregants of 21A/BY4742 having the *SMD2^21A^* allele (confirmed by genotyping the individual segregants, data not shown). We have also applied RHA for *SMD2* in the original 21A/BY4742 hybrid. Interestingly, we could not detect any difference in thermotolerance at the two temperatures tested (40.7°C and 41°C) (Figure 7A). Knowing that 21A^DG^/BY4742^DG^ lack only two superior alleles as compared to 21A/BY4742 and both *PRP42* and *SMD2* encode proteins forming the same spliceosomal complex, we constructed double hetero-allelic mutations for *PRP42* and *SMD2* in the 21A/BY4742 background, and evaluated thermotolerance of the strains. In the hybrid with the inferior *PRP42* allele, the superior *SMD2* allele caused higher thermotolerance compared to the inferior *SMD2* allele, whereas in the hybrids containing the superior *PRP42* allele, the two *SMD2* alleles did not influence thermotolerance differently (Figure 7B). The identification of *SMD2* as a causative gene for thermotolerance indicates that our new approach of mapping with the downgraded parent strains is able to reveal minor loci and causative genes that escape detection in QTL mapping with the original parents, in this specific case because of epistatic interaction.

### Expressing *PRP42^BY4742^* in 21A does not enhance its thermotolerance

We expressed the two *PRP42* alleles from a centromeric plasmid in the parent 21A strain (Figure S7A online) and in the 21A *prp42Δ* strain (Figure S7B online). In both cases, there was no difference in thermotolerance between the strains. On the other hand, comparison of the thermotolerance of strain 21A and that of the two heterozygous RHA strains showed that the RHA strain expressing the 21A allele had clearly lower thermotolerance than the other two strains (Figure S7C online). The thermotolerance of the heterozygous RHA strain expressing the superior *PRP42* allele from BY4742 was not higher than that of the 21A strain. These results show that the BY4742 allele of *PRP42* is not able to enhance the thermotolerance level of the 21A strain further, apparently indicating that other factors become limiting for thermotolerance. One such other factor may be *SMD2*. In the 21A strain it is present for 100% in the superior form, while in the heterozygous RHA strain, it is only present for 50% in the superior form. Hence, a dosage effect of *SMD2* may possibly be limiting for thermotolerance in the heterozygous RHA strain expressing the superior *PRP42* allele from BY4742. The difference in ploidy or in the genetic constitution between the haploid 21A strain and the diploid RHA hybrid strains may also play a role, although this seems to be contradicted by the fact that we mapped the superior *PRP42* allele using haploid segregants of the superior and inferior parents. Also in the study of Sinha et al. [27], replacement of the inferior allele of *MKT1* with the superior allele in the S288c strain did not cause the expected improvement in thermotolerance.

## Discussion

Identification of QTLs with minor effects on complex traits remains a difficult issue in quantitative genetics [29]. Major approaches used up to now have been fixing of major QTLs in a single parent and repeating the QTL mapping procedure either with backcrosses or regular crosses between the parents [22], [23], [30], the use of very high numbers of segregants [24], more stringent phenotyping to enhance the detectability of the minor QTLs [10] or genotyping and phenotyping single segregants [21]

In this study, we have extended the approach of fixing major QTLs to mapping by pooled-segregant whole-genome sequence analysis. In addition, we fixed a major QTL in each parent strain to create a downgraded superior and a downgraded inferior parent strain. The benefit of downgrading both parents, especially in pooled-segregant mapping, is that it keeps a large phenotypic difference between the parental strains. This makes the isolation of a sufficient number of segregants with extreme phenotype easier or at least makes the evaluation of their phenotype in comparison with that of the superior parent easier. In addition, it may enhance the chances that the minor QTLs identified are truly relevant for the phenotypic difference between the original parents and not generated in some unrelated manner in the mapping procedure. The advantages of fixing major QTLs in one parent for linkage mapping have recently been demonstrated [23]. Fixing major QTLs in both parents may have a similar advantage for linkage mapping as in pooled-segregant analysis, since it increases the potential of a wider range of phenotypic variation and thus also for more reliable selection of segregants with an extreme phenotype.

If enough phenotypic variability is obtained in the segregants, one could in principle map with parents that do not differ at all in the trait-of-interest. However, in this case one is mapping mutations in the background of the strains that affect the trait-of-interest. This is not the general purpose of our work. In our case, the goal was to identify the mutant alleles that are responsible for the elevated thermotolerance in the superior parent strain, so that these alleles after their identification could be transferred to other industrial yeast strains to enhance their thermotolerance. In principle, one could also do a second round of mapping with upgraded parents. However, we believe that mapping with downgraded parents has a higher chance of revealing additional minor QTLs because it eliminates epistatic interactions with the major QTLs and also because elimination of the major QTLs enhances requirement for the presence of minor QTLs if the screening of the phenotype is performed at a similar stringency.

Our approach is based on the observation that the causative genetic element(s) in some QTLs is(are) linked to the inferior rather than to the superior parent. This is likely due to the fact that genetic mapping in yeast is performed with haploid strains derived from natural or industrial diploid strains that generally harbor a single copy of many recessive alleles. As a result of the presence of negative, recessive mutations, positively acting QTLs and causative genes will be identified that are linked to the inferior rather than the superior parent. This has also been observed in several previous mapping studies [10], [18], [24]. It indicates that linkage of QTLs to the inferior parent is not an uncommon phenomenon and, moreover, may significantly increase when the influence of major QTLs is weakened or when genetic linkage in the genome is reduced.

Identification of the causative gene in QTL1, linked to the superior parent, and in QTL3, linked to the inferior parent, allowed us to construct both a downgraded, superior and a downgraded, inferior parent strain using targeted allele replacement. Repeating the genetic mapping with the downgraded parent strains successfully revealed new minor QTLs and thus established the effectiveness of this approach. Moreover, we validated the new QTLs 4 and 5 by identifying the causative genes. QTL4 contained a causative gene previously identified for high thermotolerance in another yeast background [22], further underscoring the effectiveness of this approach. Interestingly, our identification in the cross with the downgraded parent strains of new QTLs linked to both superior and inferior parent, allows in principle to construct further downgraded parent strains and repeat the mapping to identify additional minor QTLs with significant linkage.

An advantage of our approach is that it keeps all genetic information from both superior and inferior parents, whereas in backcrossing approaches, 50% of the genetic information of the superior parent is lost. As a result, minor QTLs may be missed. Furthermore, backcrossing creates regions that are identical between the new parents, i.e. the F1 segregant and the inferior parent, which makes it impossible to identify in the next cross QTLs linked to the inferior parent in these regions. Although the phenotype of the downgraded parent may not be as extreme as that of the F1 segregant normally selected for backcrossing, it has all the genetic diversity to generate segregants with a phenotype as extreme as obtained in the backcross.

Another advantage compared to backcrossing of repeating the QTL mapping after fixing causative genes in the parents is that it can reveal new minor QTLs and causative genes located closely to or even within the previously identified QTL. If the superior alleles that have been replaced in the downgraded parent strains were the only causative gene in their QTL, this QTL should disappear completely in the second cross. In our case, this happened with QTL3, for which there was no linkage anymore with the segregants of the downgraded parents. On the other hand, if other causative genes exist within the QTL in addition to the fixed gene, the QTL will likely remain present in the second mapping, allowing identification of the remaining causative gene(s). This happened in our case with QTL1, which shifted to a slightly more upstream position. In the new QTL, which was called QTL4, we could subsequently confirm *NCS2* as the causative gene. The presence of multiple causative genes located close to each other within a single QTL has been found before [6], [10], [22]. To resolve closely located QTLs in the first cross an impractical number of F1 segregants is easily required [31]. Recently, multiple, random inbreeding with all F1 segregants was used to enhance recombination between the genomes of the parents and thus reduce linkage in the genome. This resulted in a higher resolution of genetic mapping, facilitating detection of closely located minor QTLs and also strongly reduced the number of candidate genes in the centre of the QTL [24].

The appearance of new minor QTLs in the second mapping, with QTL5 and its causative gene *SMD2* as a striking example, raises the question why these QTLs were not detected in the first mapping. One plausible explanation is interaction between causative genes from different QTLs, which has been identified by Lorenz et al. [23]. In our study we identified a negative interaction between the *SMD2* and *PRP42* alleles, which can explain the absence of QTL5 in the first mapping. In the latter, the presence of the superior *PRP42* allele in the selected thermotolerant segregants could compensate for the presence of an inferior *SMD2* allele. In the second mapping, after removal of the superior *PRP42* allele, the effect of the superior *SMD2* allele now apparently became more significant, causing a higher chance for this allele to be present in the thermotolerant segregants.

Thermotolerance of growth, which is the ability to grow at elevated temperatures, has been a favourite trait in quantitative genetics with yeast [6], [22], [24], [27], [32], [33]. It is easily scored on solid nutrient plates, it is highly relevant for several industrial applications with yeast and is a typical characteristic of clinical isolates of *S. cerevisiae*. To date, several genes have been identified in natural yeast strains with an allele-specific contribution to thermotolerance. The QTLs identified in our study did not overlap with the regions in which these genes are located, except for QTL1 (*MKT1*) and QTL4 (*NCS2*). The diverse biological functions of these genes underscores our limited understanding of this phenotype, since apparently none of these genes has a function that can be directly linked in a known mechanistic manner to sustaining high thermotolerance.

In this study, we have identified *PRP42^BY4742^* and *SMD2^21A^* as two novel and naturally-occurring superior alleles for high thermotolerance. Yeast Prp42 was identified as an essential protein for U1 small nuclear ribonucleoprotein (snRNP) biogenesis, which has a high similarity to Prp39 [34]. *SMD2* encodes a core protein Sm D2 that is part of the spliceosomal U1, U2, U4, and U5 snRNPs [35]. These snRNPs function in pre-mRNA splicing by recognizing short conserved sequences from 5′ to 3′ at the exon-intron junctions and assemble into active spliceosomes [36]. Interestingly, the related function of these two genes suggests an important role for RNA processing in growth at high temperature. Further analysis revealed an allele-specific interaction between *PRP42* and *SMD2*. This is consistent with the previous evidence for direct interaction between the human homologues of these gene products as revealed by crystal structure determination of human spliceosomal U1 snRNP [37].

The *MKT1* gene has been found as a causative gene in several QTL mapping studies with various phenotypes and using diverse genetic backgrounds, but always with the S288c/BY background for the control parent [10], [15], [30], [38]. Mkt1 appears to control gene expression at a post-transcriptional step [39], which may explain why its deficiency produces effects on such a diversity of phenotypes.

To allow faster identification of causative genes in the mapped QTLs, we have applied bulk RHA, which evaluates multiple adjacent genes simultaneously. The successful identification of causative genes (*PRP42* and *SMD2*) using this approach confirms the effectiveness of this method. A possible advantage of this strategy over RHA with single alleles is that it can take into account genetic interactions [19] between the genes in the deleted region. If two closely located genes can compensate for each other, bulk RHA may detect their effect as opposed to single gene RHA. Another advantage of bulk RHA is its high efficiency, especially in cases where QTLs cannot be reduced to a small size with only few genes in the centre because of a limited number of segregants available for fine-mapping. In general, this will be the case with phenotypes that require a high workload for scoring. In our experience, with bulk RHA one can easily evaluate a region with a size of 20 kb, which encompasses on average between 6 and 12 genes in yeast. On the other hand, bulk RHA carries possible pitfalls. When a region used for bulk deletion carries both positively acting and negatively acting genes, as was found in previous studies [6], [10], simultaneous deletion of both can result in the absence of any phenotypic effect. Hence, a negative result with bulk RHA does not necessarily imply the absence of causative genes.

### Conclusions

In this paper we have shown that identification of new minor QTLs involved in complex traits can be successfully accomplished by crossing parent strains that have both been downgraded for a single QTL. Using this approach we have identified new QTLs and new causative genes, revealing an important role for RNA processing in high thermotolerance. This method has the advantage of maintaining all relevant genetic diversity and enough phenotypic difference between the two parent strains and thus significantly increases the chances of identifying minor QTLs. In principle, successive rounds of minor QTL mapping could be performed in this way by sequentially downgrading the two parent strains further, making use each time of a causative gene identified in a QTL linked to the superior parent and in a QTL linked to the inferior parent.

## Materials and Methods

### Yeast strains, growth conditions and sporulation

The following yeast strains were used: prototrophic and heterothallic diploid strain MUCL28177, which was isolated from orange juice in the region of Strombeek-Bever, Belgium, its haploid segregant MUCL28177-21A, referred to as 21A, and BY4742 (*Matα his3Δ1 leu2Δ0 ura3Δ0 lys2Δ0*) [40]. Yeast cells were grown in YPD medium containing 1% (w/v) yeast extract, 2% (w/v) bacteriological peptone, and 2% (w/v) glucose. 1.5% (w/v) Bacto agar was used to make solid nutrient plates. Transformants were grown on YPD agar plates containing 200 µg/ml geneticin. Mating, sporulation and isolation of haploid segregants were done using standard protocols [41].

### Phenotyping

Strains were inoculated in liquid YPD and grown in a shaking incubator at 30°C overnight. The next day the cells were transferred to fresh liquid YPD at an OD_600_ of 1 and grown for 2 to 4 h to enter exponential phase. The cell cultures were then diluted to an OD_600_ of 0.5 and 5 µl of a fourfold dilution range was spotted on YPD agar plates, which were incubated at different temperatures. Growth was scored after two days incubation for all conditions. All spot tests were repeated at least once, starting with freshly inoculated cultures. Repetitions of the thermotolerance assays may show slight differences in growth intensity. Hence, the strains to be tested were always spotted together with the relevant controls on the same plate.

### Pooled-segregant whole-genome sequence analysis and determination of SNP variant frequency

#### Whole-genome sequencing

For each genetic mapping experiment, 58 thermotolerant segregants were grown separately in 50 ml liquid YPD cultures at 30°C for three days. Cell dry weight was measured for each culture and the cultures were pooled based on the same dry weight. Genomic samples of the pooled culture, together with that of 21A were isolated with standard methods [42]. At least 5 µg of each DNA sample was provided to GATC Biotech AG or BGI for sequencing. Paired-end short reads of 100 bp were generated. Sequence alignment was performed using SeqMan NGen. Assembly and mapping were done with DNAstar Lasergene.

#### Filtering

SNPs were selected for high quality, based on filtering for sufficient coverage (≥20 times) and ratio (≥80%) [10], [25]. The coverage of at least 20 times was based on previous findings that a 20-fold sequencing coverage is sufficient to compensate for errors by the number of correct reads [43]. The ratio of at least 80% was chosen based on the plots of the SNPs between the two parent strains, as described previously [10], [25].

#### Statistical model

Swinnen et al. [10] and Claesen et al. [25] developed an additive logistic regression model for a joint analysis of bulk sequencing data from different pools. They proposed to use simultaneous confidence bands to test for (a) deviations from random segregation (SNP frequency of 50%) or (b) differences between pools along the chromosome, while accounting for multiple testing. In this contribution, we extend the simulation-based inference approach of Claesen et al. [25] and provide adjusted p-values that account for multiple testing. The confidence bands and p-values in the manuscript are based on one million Monte Carlo simulations. Details on the procedure can be found in Supplementary Information: Supplementary Methods.

#### Contrast between pools

We also sequenced a pool of unselected segregants, which is referred to as pool 0 and for which random segregation can be expected. Figure S5 shows patterns in the SNP frequency profile of the unselected pool, which remind of wave effects found in copy number variation profiling [44]. A similar approach to correct for wave patterns has been adopted for bulk segregant sequencing: instead of inferring on deviations from the SNP frequency of 50% (log odds = 0), log odds ratio's between the selected pools and the unselected pool are assessed.

#### Testing against a biological threshold

Testing if the true log odds ratio between pool q and the unselected pool 0 is different from zero results in statistical significance, but cannot assure that the detected differences are large enough to be biologically meaningful. Following McCarthy and Smyth [45], we test relative to a biological relevant threshold δ for ensuring both statistical significance and biological relevance. The threshold is chosen at δ = 0.4088. This is equivalent to testing if the odds ratio of pool q and pool 0 is outside the interval [2/3,3/2], e.g. it corresponds to testing if the SNP frequency for pool q is outside [40%, 60%] when the SNP frequency of pool 0 equals 50%.

### SNP scoring in individual segregants

SNPs were scored in individual segregants by PCR. At a given chromosomal location, two SNPs spacing between 500 and 1,500 bp were chosen for the design of specific primers. For a given SNP, two primers either in the forward or reverse direction, were designed with one mismatch at their 3′ ends. First, a gradient PCR was applied using genomic samples of 21A and BY4742 as templates, with each template tested with two primer combinations (primer pair based on the sequence of BY4742 and primer pair based on the sequence of 21A). The annealing temperature at which the best distinguishing power was obtained with the two parents was used for scoring of the SNPs in the individual segregants.

#### Statistical analysis

The SNP data in the individual segregants have been analysed using the binomial exact test. The p-values have been adjusted for multiple testing under dependency using the Benjamini Yekutieli False Discovery Rate (FDR) method [26].

### Reciprocal hemizygosity analysis

All the ORFs of non-essential genes in the centre of the QTL were deleted separately in both 21A and BY4742. PCR-mediated gene disruption was used [46]. Plasmid pFA6a was used as a template to amplify a linear DNA fragment containing the kanMX4 cassette [47], with 50 bp homologous sequences for the target regions at both ends. Transformants growing on YPD geneticin plates were verified by PCR with several combinations of internal and external primers. The verified haploid deletion strains were subsequently crossed with the matching wild type haploid to generate the hybrid diploids. For RHA with essential genes and fragments containing multiple genes, transformation was performed directly in the hybrid diploid. External SNPs primer pairs together with internal primers within the kanMX4 cassette were used in different combinations to determine in which parent the allele or the fragment had been deleted. For each heterozygous deletion hybrid, at least two isogenic strains were made and evaluated for thermotolerance. The growth of strains in the RHA test should always be compared within the strain pairs and not between the strain pairs, since the loss of one copy of a gene can cause an effect on the growth of the strains under non-restrictive conditions or even under restrictive conditions if the gene is important for the phenotype and because of the variability between different thermotolerance assays..

### Allele replacement

The replacement of *MKT1^21A^* with *MKT1^BY4742^* in 21A was performed by a two step transformation. For the first transformation, a linear DNA fragment with the *AMD1* gene from *Zygosaccharomyces rouxii* flanked by 50 bp sequences that are homologous to the two sides of the *MKT1* ORF was amplified from plasmid pFA6a-AMD1-MX6 [48] by PCR, and transformed into 21A. Transformants were grown on YCB (Yeast Carbon Base 1.17%, phosphate buffer 3%, Bacto agar 2%) plates containing 10 mM acetamide. Single colonies were checked for the correct replacement with the use of external and internal primers. For the second transformation, colonies were transformed with a linear DNA fragment containing the *MKT1^BY4742^* ORF, together with ∼100 bp downstream and upstream. Transformants were grown on YNB galactose (0.17 Yeast Nitrogen Base w/o amino acids and ammonium sulfate, 1.5% Difco agar, 0.01% galactose, pH 6.5) containing 100 mM fluoroacetamide. Colonies were first checked for the presence of *MKT1* by PCR, and then confirmed by DNA sequencing.

The replacement of *PRP42^BY4742^* with *PRP42^21A^* in BY4742 was performed in a two step transformation. For the first transformation, a *URA3* gene was inserted ∼50 bp downstream of the *PRP42* ORF in BY4742. Colonies growing on –URA plates were confirmed to have a correct insertion by PCR. For the second transformation, a linear DNA fragment containing the ORF of *PRP42^21A^* together with ∼400 bp downstream and upstream was transformed into the previous colonies, and the transformants were grown on 5-FOA plates. Colonies were first checked for the right DNA polymorphism by SNP primer pairs, and then confirmed by DNA sequencing.

### Data access

All sequence data have been deposited in the Sequence Read Archive (SRA) at the National Center for Biotechnology Information (NCBI) and can be accessed with account number SRA058979.

## Supporting Information

Figure S1 RHA for the negative candidate genes in QTL1. For none of these candidate genes there was a clear reproducible difference in thermotolerance between the two hybrid diploids expressing a single copy of the two parental alleles.
(PDF)

Figure S2 Effect of *MKT1* deletion on thermotolerance. The *MKT1* gene was deleted in the 21A superior parent strain and in the BY4742 inferior parent strain. Deletion of *MKT1* in the 21A background caused the same drop in thermotolerance as introduction of the BY allele of *MKT1*. In the BY4742 background, deletion of *MKT1* did not affect thermotolerance All strains were spotted on the same plate and incubated at 40.7°C.
(PDF)

Figure S3 RHA for the remaining candidate genes within *FRAGMENT1* of QTL3. For none of these candidate genes there was a clear reproducible difference in thermotolerance between the two hybrid diploids expressing a single copy of the two parental alleles.
(PDF)

Figure S4 RHA with *FRAGMENT1* and *PRP42* of QTL3. *FRAGMENT1* and *PRP42* from the BY background cause a similar increase in thermotolerance compared to *FRAGMENT1* and *PRP42* from the 21A background, suggesting that *PRP42* is the main causative gene in *FRAGMENT1*. All strains were spotted on the same plate and incubated at 41°C.
(PDF)

Figure S5 Plot of the SNP variant frequency against the SNP chromosomal position for the pool of unselected segregants. The genomic DNA of the pool of 58 unselected segregants from the hybrid strain 21A^DG^/BY4742^DG^ was sequenced and analyzed in the same way as for the selected segregants. The top-panel represents the SNP variant frequency (small gray circles) along with the smoothed SNP frequency profile (black line) using an additive logistic regression model. In the middle panel the log odds of the SNP variant frequency is plotted for pool 0 along with simultaneous 95% confidence bands (gray regions). The bottom panel shows 2-sided p-values along the chromosome that are corrected for multiple testing. The SNP variant frequency only shows random variation throughout the genome.
(PDF)

Figure S6 RHA for the candidate causative gene *NCS2* in the new QTL4 identified with the downgraded parents. *NCS2^21A^* conferred higher thermotolerance than *NCS2^BY4742^*, confirming *NCS2* as causative gene in QTL4. Deletion of *NCS2^BY4742^* in 21A^DG^/BY4742^DG^ also reduced thermotolerance, indicating that *NCS2^BY4742^* is not a non-functional allele.
(PDF)

Figure S7 Expression of *PRP42* alleles in 21A. (A) *PRP42* alleles were expressed from a centromeric plasmid in 21A. (B) *PRP42* alleles were expressed from a centromeric plasmid in 21A *prp42Δ.* (C) Growth of 21A and the RHA pair for *PRP42* on the same plate.
(PDF)

Table S1 List of putative QTLs for both original and downgraded parents.
(DOCX)

Table S2 Presence of the *PRP42^21A^* ORF SNPs in other yeast strains with various origins.
(DOCX)

Table S3 *SMD2* expression analysis.
(DOCX)

Text S1 Supplementary methods.
(PDF)
