# Supplementary figures and images for: QTL Analysis of High Thermotolerance with Superior and Downgraded Parental Yeast Strains Reveals New Minor QTLs and Converges on Novel Causative Alleles Involved in RNA Processing
Source: PLoS Genet. 2013 Aug 15;9(8):e1003693. doi: 10.1371/journal.pgen.1003693 (PMC3744412; doi:10.1371/journal.pgen.1003693)

Figure S1

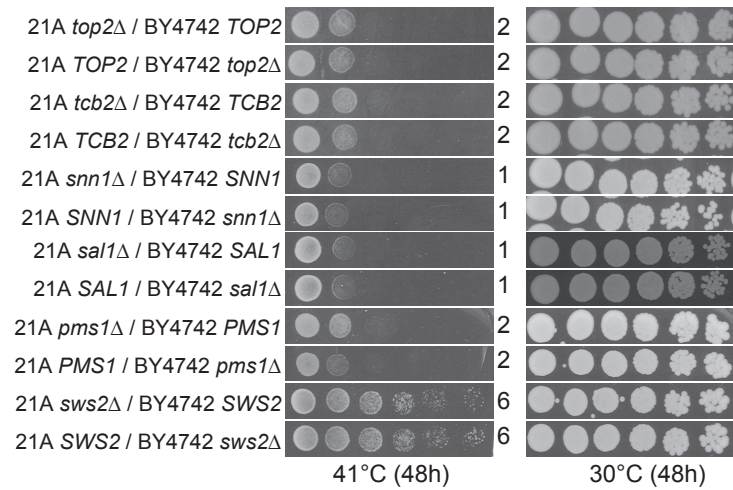

Supplement: Figure S1 — RHA for the negative candidate genes in QTL1. For none of these candidate genes there was a clear reproducible difference in thermotolerance between the two hybrid diploids expressing a single copy of the two parental alleles. (PDF) [file pgen.1003693.s001.pdf]

**Figure S2**

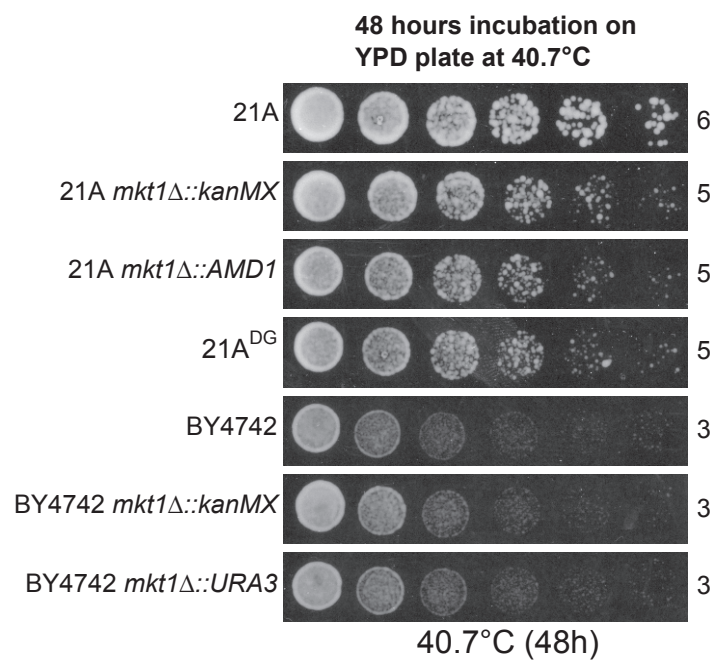

Supplement: Figure S2 — Effect of MKT1 deletion on thermotolerance. The MKT1 gene was deleted in the 21A superior parent strain and in the BY4742 inferior parent strain. Deletion of MKT1 in the 21A background caused the same drop in thermotolerance as introduction of the BY allele of MKT1. In the BY4742 background, deletion of MKT1 did not affect thermotolerance All strains were spotted on the same plate and incubated at 40.7°C. (PDF) [file pgen.1003693.s002.pdf]

Figure S3

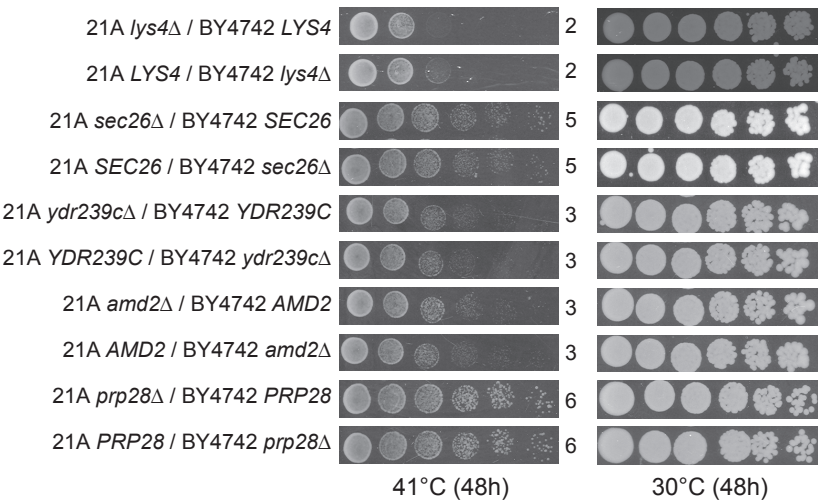

Supplement: Figure S3 — RHA for the remaining candidate genes within FRAGMENT1 of QTL3. For none of these candidate genes there was a clear reproducible difference in thermotolerance between the two hybrid diploids expressing a single copy of the two parental alleles. (PDF) [file pgen.1003693.s003.pdf]

Figure S4

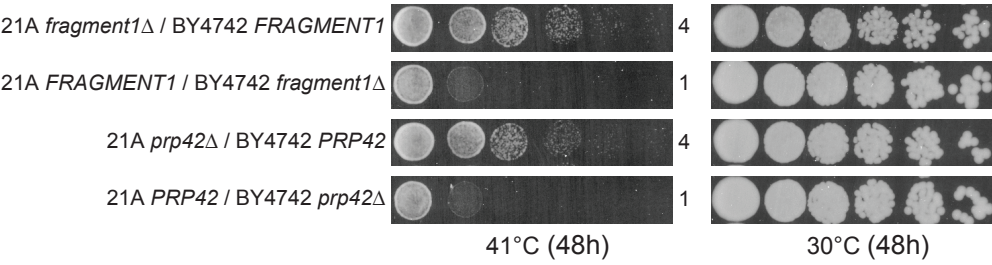

Supplement: Figure S4 — RHA with FRAGMENT1 and PRP42 of QTL3. FRAGMENT1 and PRP42 from the BY background cause a similar increase in thermotolerance compared to FRAGMENT1 and PRP42 from the 21A background, suggesting that PRP42 is the main causative gene in FRAGMENT1. All strains were spotted on the same plate and incubated at 41°C. (PDF) [file pgen.1003693.s004.pdf]

Figure S5

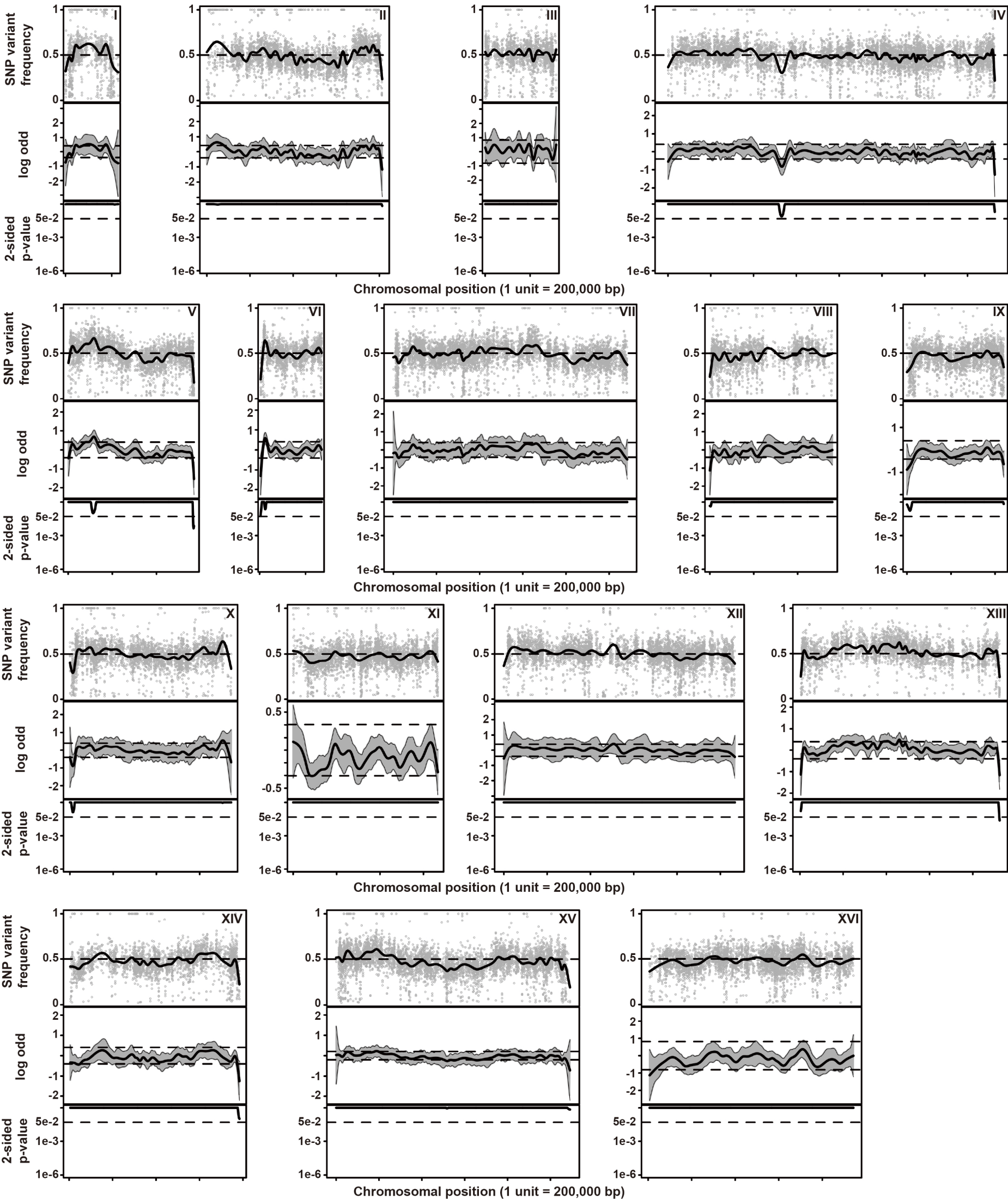

Supplement: Figure S5 — Plot of the SNP variant frequency against the SNP chromosomal position for the pool of unselected segregants. The genomic DNA of the pool of 58 unselected segregants from the hybrid strain 21ADG/BY4742DG was sequenced and analyzed in the same way as for the selected segregants. The top-panel represents the SNP variant frequency (small gray circles) along with the smoothed SNP frequency profile (black line) using an additive logistic regression model. In the middle panel the log odds of the SNP variant frequency is plotted for pool 0 along with simultaneous 95% confidence bands (gray regions). The bottom panel shows 2-sided p-values along the chromosome that are corrected for multiple testing. The SNP variant frequency only shows random variation throughout the genome. (PDF) [file pgen.1003693.s005.pdf]

Figure S6

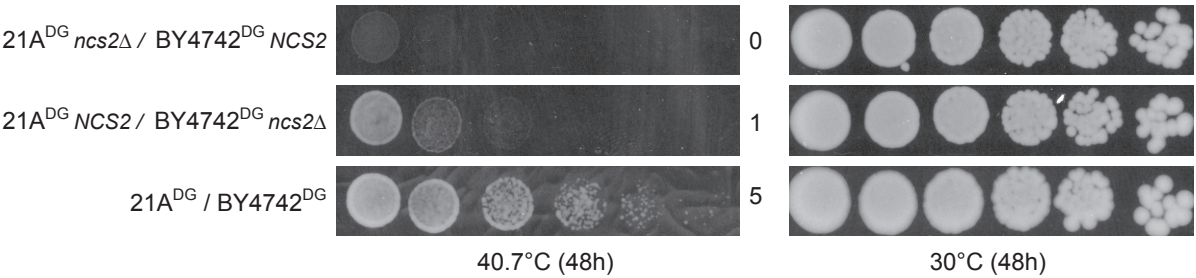

Supplement: Figure S6 — RHA for the candidate causative gene NCS2 in the new QTL4 identified with the downgraded parents. NCS221A conferred higher thermotolerance than NCS2BY4742, confirming NCS2 as causative gene in QTL4. Deletion of NCS2BY4742 in 21ADG/BY4742DG also reduced thermotolerance, indicating that NCS2BY4742 is not a non-functional allele. (PDF) [file pgen.1003693.s006.pdf]

Figure S7

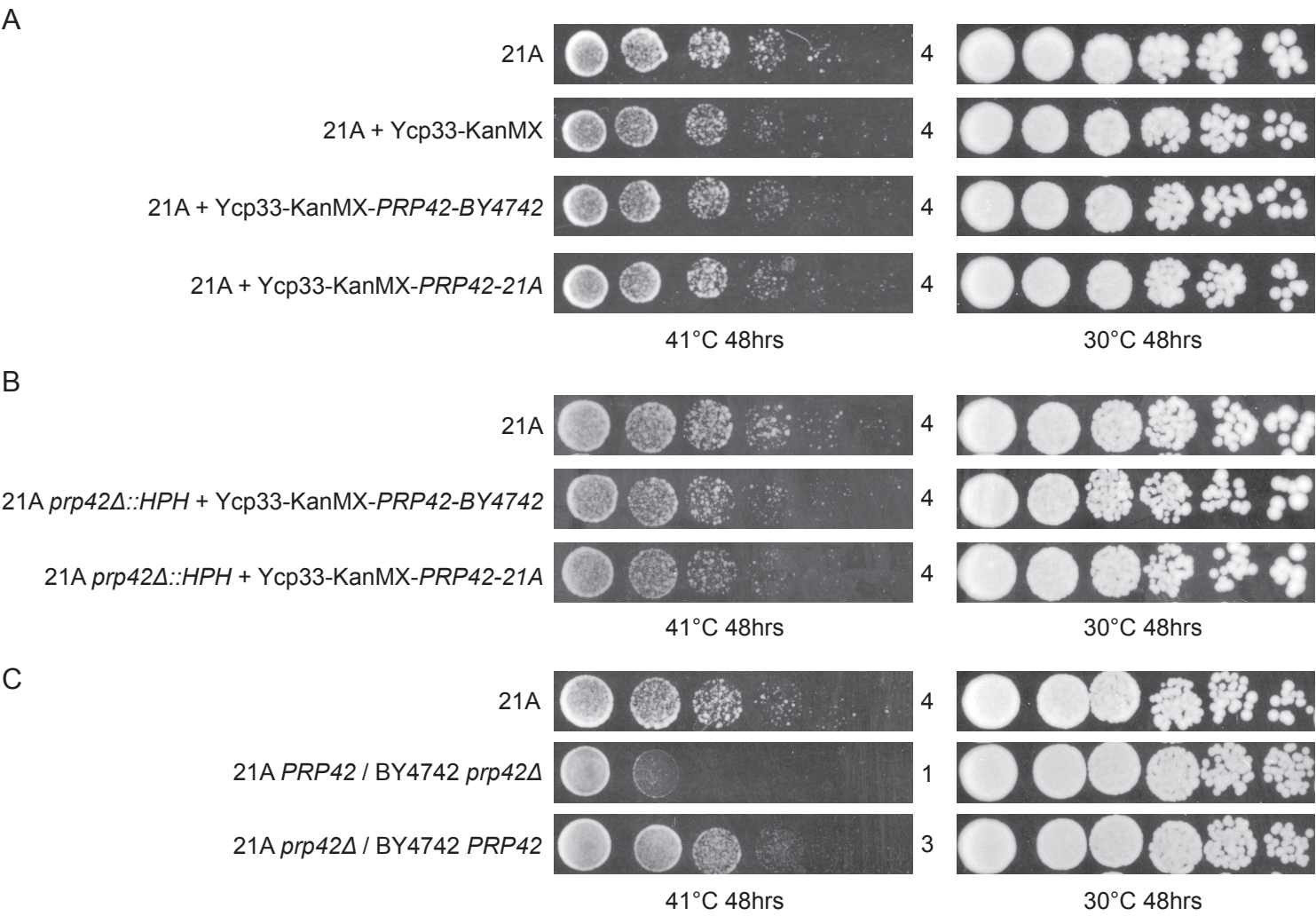

Supplement: Figure S7 — Expression of PRP42 alleles in 21A. (A) PRP42 alleles were expressed from a centromeric plasmid in 21A. (B) PRP42 alleles were expressed from a centromeric plasmid in 21A prp42Δ. (C) Growth of 21A and the RHA pair for PRP42 on the same plate. (PDF) [file pgen.1003693.s007.pdf]
