# Supplementary material for: QTL Analysis of High Thermotolerance with Superior and Downgraded Parental Yeast Strains Reveals New Minor QTLs and Converges on Novel Causative Alleles Involved in RNA Processing
Source: PLoS Genet. 2013 Aug 15;9(8):e1003693. doi: 10.1371/journal.pgen.1003693 (PMC3744412; doi:10.1371/journal.pgen.1003693)
Supplement: Table S1 — List of putative QTLs for both original and downgraded parents. (DOCX) [file pgen.1003693.s008.docx]

**Table S1. List of putative QTLs from the mapping with both the original and downgraded parents**

| **QTL** | **Location** | **Total number of thermotolerant segregants used** | **Predicted association to superior parent strain 21A** | **2-sided p-value** |
| --- | --- | --- | --- | --- |
| **QTLs identified with the original parents** | | | | |
| QTL1^*^ | 366505-584530 on chromosome XIV | 58 | 98.7% | <1e-6 |
| QTL2^*^ | 513989-595289 on chromosome II | 58 | 70.8% | 2.3e-4 |
| QTL3^*^ | 913355-974438 on chromosome IV | 58 | 31.7% | 7.0e-3 |
| putative QTL^**^ | 600000 on chromosome XIII | 58 | 70.5% | 11e-3 |
| rejected QTL^***^ | 559076-601738 on chromosome XVI | 58 | 67.4% | 1.8e-3 |
| **QTLs identified with the downgraded parents** | | | | |
| QTL4^*^ | 330686-492648 on chromosome XIV | 58 | 90.1% | <1e-6 |
| QTL5^*^ | 636083-794021 on chromosome XII | 58 | 83.3% | 1.5e-4 |
| Putative QTL^**^ | 538379-644069 on chromosome II | 58 | 79.4% | 2.0e-6 |
| Putative QTL^**^ | 263962-300626 on chromosome III | 58 | 25.0% | 1.2e-6 |
| Putative QTL^**^ | 240444-388306 on chromosome V | 58 | 71.8% | 1.4e-4 |
| Putative QTL^**^ | 195634-202548 on chromosome VI | 58 | 31.7% | 3.9e-6 |
| Putative QTL^**^ | 607273-674469 on chromosome VII | 58 | 27.5% | 1.3e-6 |
| Putative QTL^**^ | 335879-410467 on chromosome XI | 58 | 25.7% | 5.7e-5 |
| Putative QTL^**^ | 672216-714549 on chromosome XIII | 58 | 74.3% | 7.1e-5 |
| Putative QTL^**^ | 407121-420274 on chromosome XVI | 58 | 28.5% | 3.1e-2 |
|  |  |  |  |  |
|  |  |  |  |  |

* Linkage was confirmed by a binomial exact test with FDR adjusted p-value.

** Linkage has not been confirmed by a binomial exact test.

*** Linkage was rejected by a binomial exact test with FDR adjusted p-value.
