# Supplementary material for: QTL Analysis of High Thermotolerance with Superior and Downgraded Parental Yeast Strains Reveals New Minor QTLs and Converges on Novel Causative Alleles Involved in RNA Processing
Source: PLoS Genet. 2013 Aug 15;9(8):e1003693. doi: 10.1371/journal.pgen.1003693 (PMC3744412; doi:10.1371/journal.pgen.1003693)
Supplement: Table S2 — Presence of the PRP4221A ORF SNPs in other yeast strains with various origins. (DOCX) [file pgen.1003693.s009.docx]

**Table S2. PRP42^21A^ mutations in other yeast strains with various origins**

|  | 855 | 861 | 886 * | 936 | 1005 | 1400 * | 1410 | 1509 * | 1576 | 1596 | 1635 |
| --- | --- | --- | --- | --- | --- | --- | --- | --- | --- | --- | --- |
| 21A | C | T | T | G | G | C | A | G | C | A | A |
| BY4742 | T | C | C | A | A | T | G | A | G | G | G |
| CEN.PK113-7D | T | C | C | A | A | T | G | A | G | G | G |
| W303 | T | C | C | A | A | T | G | A | G | G | G |
| FosterO | T | C | C | A | G | C | A | G | C | G | A |
| VL3 | T | C | C | A | A | C | G | G | C | G | A |
| M22 | T | C | C | A | A | C | G | G | C | G | A |
| CLIB215 | T | C | C | A | A | C | G | G | C | G | A |
| JAY291 | T | C | C | A | A | C | A | G | C | G | A |
| AWRI1631 | T | C | C | A | A | C | G | G | C | G | A |
| EC1118 | C | T | C | A | G | C | A | G | C | A | A |
| Kyokai7 | T | C | C | A | A | C | A | G | C | A | A |
| YJM269 | T | C | C | A | A | C | A | G | C | A | A |
| CBS1585-Seg5 | C | T | C | A | G | C | A | G | C | A | A |
| UC5 | C | T | C | A | G | C | A | G | C | A | A |
| YPS163 | C | T | C | A | G | C | A | G | C | A | A |
| Sigma1278b | C | T | C | A | G | C | A | G | C | G | A |
| ZTW1 | C | T | C | A | G | C | A | G | C | G | A |
| YJM789 | C | T | C | A | G | C | A | G | C | G | A |
| ER18 | C | T | C | A | G | C | A | G | C | G | A |
| PW5 | C | T | C | A | G | C | A | G | C | A | A |
| T7 | C | T | C | G | G | C | A | G | C | A | A |
| FosterB | T | C | C | A | G | C | A | G | C | G | A |
| AWRI796 | T | C | C | A | A | C | G | G | C | G | A |

* non-synonymous mutations
