# Supplementary material for: QTL Analysis of High Thermotolerance with Superior and Downgraded Parental Yeast Strains Reveals New Minor QTLs and Converges on Novel Causative Alleles Involved in RNA Processing
Source: PLoS Genet. 2013 Aug 15;9(8):e1003693. doi: 10.1371/journal.pgen.1003693 (PMC3744412; doi:10.1371/journal.pgen.1003693)
Supplement: Table S3 — SMD2 expression analysis. (DOCX) [file pgen.1003693.s010.docx]

**Table S3. Expression analysis of *SMD2***

|  | **6 hours culture at 30 °C** | | **6 hours culture at 30°C**  **+ 1 hour at 40.7°C** | |
| --- | --- | --- | --- | --- |
|  | ***SMD2* relative expression *** | **SD** | ***SMD2* relative expression *** | **SD** |
| 21A *smd2Δ* / BY4742 *SMD2* | 1.00 | 0.03 | 0.51 | 0.04 |
| 21A *SMD2* / BY4742 *smd2Δ* | 2.84 | 0.12 | 1.14 | 0.04 |
| 21A *prp42Δ smd2Δ* / BY4742 *PRP42 SMD2* | 1.28 | 0.04 | 0.53 | 0.04 |
| 21A *prp42Δ SMD2* / BY4742 *PRP42 smd2Δ* | 1.52 | 0.06 | 1.51 | 0.07 |
| 21A *PRP42 smd2Δ* / BY4742 *prp42Δ SMD2* | 0.89 | 0.03 | 0.58 | 0.05 |
| 21A *PRP42 SMD2* / BY4742 *prp42Δ smd2Δ* | 1.13 | 0.08 | 0.54 | 0.02 |
| 21A^DG^ *smd2Δ* / BY4742^DG^ *SMD2* | 1.22 | 0.11 | 0.73 | 0.02 |
| 21A^DG^ *SMD2* / BY4742^DG^ *smd2Δ* | 1.78 | 0.06 | 0.88 | 0.04 |
| 21A ** | 1.79 | 0.16 |  |  |
| BY4742 ** | 1.00 | 0.13 |  |  |
| 21A^DG^ ** | 1.48 | 0.16 |  |  |
| BY4742^DG^ ** | 0.72 | 0.08 |  |  |

* Relative levels to those of *UBC6* and *TFC1*

** Expression analysis with the haploid strains was performed in a separate experiment
