# Supplementary material for: QTL Analysis of High Thermotolerance with Superior and Downgraded Parental Yeast Strains Reveals New Minor QTLs and Converges on Novel Causative Alleles Involved in RNA Processing
Source: PLoS Genet. 2013 Aug 15;9(8):e1003693. doi: 10.1371/journal.pgen.1003693 (PMC3744412; doi:10.1371/journal.pgen.1003693)
Supplement: Text S1 — Supplementary methods. (PDF) [file pgen.1003693.s011.pdf]

Text S1 Supplementary Methods

Claesen et al. (2013) developed an additive model for analyzing bulk sequencing data of Q different pools, simultaneously.

$$\begin{cases} \mathrm{E}[Y_{iq}] & = & n_{iq}\pi_{iq} \\ \mathrm{Var}[Y_{iq}] & = & n_{iq}\pi_{iq}(1-\pi_{iq})\phi \\ \mathrm{logit}(\pi_{iq}) & = & f_q(x_i) \end{cases} \quad (1)$$

with Y_{iq} the number of reads that cover the ith SNP and share the nucleotide of the parent with the trait, n_{iq} the total number of reads for pool q that cover the ith SNP, π_{iq} the segregation frequency for pool q at SNP i, ϕ the dispersion parameter to model overdispersion with respect to the binomial distribution, the logit function $\mathrm{logit}(\pi_{iq}) = \log[\pi_{iq}/(1-\pi_{iq})]$ is the log odds of π_{iq}, x_i the chromosomal location of SNP i and $f_q(x_i)$ the log odds of the SNP frequency at position x_i. The two first moments of Y are modelled: the mean $\mathrm{E}[Y_{iq}]$ and the variance $\mathrm{Var}[Y_{iq}]$. The method allows to draw inference on individual segregation frequency profiles $f_q(.)$ as well as on on contrasts $f_q(.) - f_r(.)$. In the remainder we will indicated the profile/contrast that will be used for statistical inference by the function $g(.)$. The method of Claesen et al. (2013) provides an estimator for $g(.)$, $\hat{g}(.)$ along with a standard error $se_{\hat{g}}(.)$. They proposed to use confidence bands for statistical inference. Asymptotic point-wise confidence bands at a specific location x can be constructed by

$$[L, U] = [\hat{g}(x) - t_{1-\alpha/2}se_{\hat{g}}(x), \hat{g}(x) + t_{1-\alpha/2}se_{\hat{g}}(x)], \quad (2)$$

with L (U) the lower (upper) bound of the interval and $t_{1-\alpha/2}$ a critical value from the standard normal distribution. When one wants to infer on a set of positions $x \in \mathcal{X}$, e.g. all locations x of a particular chromosome, point-wise confidence bands can no longer be used as they cannot control the probability

$$P\{L(x) \leq f(x) \leq U(x) \text{ for } \forall x \in \mathcal{X}\} \leq 1 - \alpha.$$

Hence, the confidence bands have to be altered for simultaneous inference on all positions in set $\mathcal{X}$. Simultaneous confidence bands can assess the null hypothesis of random segregation at any position along the curve while correcting for the multiple comparison problem. Ruppert et al. (2002, sec. 6.5) extend traditional pointwise confidence bands to simultaneous confidence bands by replacing the critical value $t_{1-\alpha/2}$ with an adjusted critical value $m_{1-\alpha}$:

$$[\hat{g}(x) - m_{1-\alpha}se_{\hat{g}}(x), \hat{g}(x) + m_{1-\alpha}se_{\hat{g}}(x)]. \quad (3)$$

In the Ruppert method, $m_{1-\alpha}$, is derived from the distribution $G(m)$ of

$$m = \sup_{x \in \mathcal{X}} \left| \frac{\hat{g}(x) - g(x)}{se_{\hat{g}}(x)} \right|,$$

where sup stands for the supremum operator and $|.|$ is the absolute value operator. Hence, $G(m)$ is the distribution of the maximum standardized difference of the estimated curve and the true curve, and, $m_{1-\alpha}$ is its $(1-\alpha)$-quantile. Similar to Ruppert et al. (2002) the distribution of $G(m)$ is estimated using a simulation based approach. In our application, $m_{1-\alpha}$ is typically 3-4 times larger than the critical value $t_{1-\alpha/2}$ from the standard normal distribution.

The simultaneous confidence bands based on $m_{1-\alpha}$ can also be used for statistical hypothesis testing at the significance level α. In this contribution the null hypothesis of random segregation $(H_0 : g(x) = 0)$ is assessed along the chromosome $(x \in \mathcal{X})$. Hence, the set of genomic locations that is significant at the significance level α is given by

$$\{\forall x : 0 \notin [\hat{g}(x) - m_{1-\alpha}se_{\hat{g}}(x), \hat{g}(x) + m_{1-\alpha}se_{\hat{g}}(x)]\},$$

the locations for which the zero-line is located outside the simultaneous confidence bands. Note, that $g(x)$ is defined on the logit scale. On the one hand, $g(x) = 0$ corresponds to a log odds of

0 or a SNP frequency of 50% when $g(.)$ is a single SNP frequency profile. On the other hand it corresponds to a log odds ratio of 0 when $g(.)$ is the contrast between the profile for pool q and the unselected pool 0, stating that there is no difference in SNP frequency between the selected pool q and the unselected pool 0 on chromosomal location x.

Instead of providing inference at the nominal significance level α the simulated distribution $\hat{G}(m)$ can also be used for deriving an adjusted p-value at every position x, p(x):

$$p(x) = \text{argmax}\left\{p(x) : 0 \notin [\hat{g}(x) - m_{1-p(x)} se_{\hat{g}}(x), \hat{g}(x) + m_{1-p(x)} se_{\hat{g}}(x)]\right\},$$

so for every location x we find the percentile $[1 - p(x)]$ of the simulated distribution $G(m)$ that gives the widest confidence band that does not contain the zero-line.

The test above assesses if $g(x)$ is significantly different from zero, but does not assure that the discovered significant differences are large enough to be biologically meaningful. Similar to McCarthy and Smyth (2009) we test relative to a threshold δ and use a composite null hypothesis $H_0 : -\delta \leq g(x) \leq \delta$. The composite null hypothesis can also be tested using simultaneous confidence bands and the set of genomic locations that is significant at the significance level α is given by

$$\{\forall x : [-\delta, \delta] \cap [\hat{g}(x) - m_{1-\alpha} se_{\hat{g}}(x), \hat{g}(x) + m_{1-\alpha} se_{\hat{g}}(x)] = \emptyset\},$$

the locations for which the confidence bands do not overlap with the H_0-region. Again, the simulated distribution $\hat{G}(m)$ can be used for deriving an adjusted p-value at every position x.

References

Claesen, J., Clement, L., Shkedy, Z., Foulqui-Moreno, M. and T. Burzykowski (2013). Simultaneous Mapping of Multiple Gene Loci with Pooled Segregants. PLoS ONE 8(2): e55133.

McCarthy, D. J. and G. K. Smyth (2009). Testing significance relative to a fold-change threshold is a TREAT, Bioinformatics, 25, 765–771.

Ruppert, D., Wand, M. and R. Carroll (2003). Semiparametric regression. Cambridge University Press, 386p.
